# Novel Non-phosphorylated Serine 9/21 GSK3β/α Antibodies: Expanding the Tools for Studying GSK3 Regulation

**DOI:** 10.3389/fnmol.2016.00123

**Published:** 2016-11-17

**Authors:** Tessa Grabinski, Nicholas M. Kanaan

**Affiliations:** ^1^Department of Translational Science and Molecular Medicine, College of Human Medicine, Michigan State University, Grand RapidsMI, USA; ^2^Hauenstein Neuroscience Center, Mercy Health Saint Mary’s, Grand RapidsMI, USA

**Keywords:** glycogen synthase kinase, signal transduction, kinase, phosphorylation, monoclonal antibody

## Abstract

Glycogen synthase kinase 3 (GSK3) β and α are serine/threonine kinases involved in many biological processes. A primary mechanism of GSK3 activity regulation is phosphorylation of N-terminal serine (S) residues (S9 in GSK3β, S21 in GSK3α). Phosphorylation is inhibitory to GSK3 kinase activity because the phosphorylated N-terminus acts as a competitive inhibitor for primed substrates. Despite widespread interest in GSK3 across most fields of biology, the research community does not have reagents that specifically react with nonphosphoS9/21 GSK3β/α (the so-called “active” form). Here, we describe two novel monoclonal antibodies that specifically react with nonphosphoS9/21 GSK3β/α in multiple species (human, mouse, and rat). One of the antibodies is specific for nonphospho-S9 GSK3β (clone 12B2) and one for nonphospho-S9/21 GSK3β/α (clone 15C2). These reagents were validated for specificity and reactivity in several biochemical and immunochemical assays, and they show linear detection of nonphosphoS GSK3. Finally, these reagents provide significant advantages in studying GSK3β regulation. We used both antibodies to study the regulation of S9 phosphorylation by Akt and protein phosphatases. We used 12B2 (due to its specificity for GSK3β) and to demonstrate that protein phosphatase inhibition reduces nonphospho-S9 GSK3β levels and lowers kinase activity within cells. The ability to use the same reagent across biochemical, immunohistological and kinase activity assays provides a powerful approach for studying serine-dependent regulation of GSK3β/α.

## Introduction

Glycogen synthase kinase (GSK) was described in the 80’s for its role in regulating glycogen synthase (Embi et al., 1980; Rylatt et al., 1980). GSK3 is a highly conserved serine (S)/threonine (T) kinase and two genes encode the α and β paralogs. The primary consensus sequence of GSK3β substrates is S/T-X-X-X-pS/pT, where phosphorylation of the target S/T is facilitated by a “priming” phosphate group on a S or T four amino acids downstream (Forde and Dale, 2007; Medina and Wandosell, 2011). The primed residue binds the primed substrate pocket bringing the target S/T residue into the kinase domain (Beurel et al., 2015). A number of GSK3β substrates such as glycogen synthase, β-catenin and tau are phosphorylated by GSK3β after being primed by other kinases (Zhang et al., 1993; Hagen and Vidal-Puig, 2002; Li et al., 2006), but not all GSK3β substrates require priming (Wang et al., 1994b).

Over 80 proposed GSK3 targets exist (most of which are not exclusive GSK3 substrates) and this kinase plays a role in numerous biological processes such as cell proliferation, cell migration, cell polarization, transcription, glucose regulation, the immune system, and inflammation (Frame and Cohen, 2001; Kim and Kimmel, 2006; Kockeritz et al., 2006; Sutherland, 2011; Beurel et al., 2015). Canonical Wnt signaling, where it phosphorylates β-catenin to signal the degradation of β-catenin, is a well-studied GSK3β pathway (Aberle et al., 1997; Chen et al., 2000; McManus et al., 2005). GSK3β also is involved in apoptosis, cancer biology, and several neurodegenerative diseases (Hetman et al., 2000; Hernandez and Avila, 2008; Cadigan and Peifer, 2009; Golpich et al., 2015; Li et al., 2015; O’Leary and Nolan, 2015). With such diverse functions, it is not surprising that GSK3 activity is tightly regulated.

In general, phosphorylation at S9 in GSK3β or S21 in GSK3α by other kinases and/or autophosphorylation is the prominent regulatory mechanism, but several additional regulatory mechanisms exist (Beurel et al., 2015). S9/S21 phosphorylation leads to inactivation because the N-terminus of GSK3 competitively blocks substrate docking in the primed substrate pocket (Frame et al., 2001) acting as a dominant negative regulator of GSK3 activity, especially against substrates requiring priming. *In situ*, GSK3 is regulated, at least in part, by phosphorylation at S9 from Akt leading to reduced activity (Gold et al., 2000; Varea et al., 2010; Majewska and Szeliga, 2016) and protein phosphatases that dephosphorylate S9 leading to increased activity under several biological contexts (Sutherland et al., 1993; Leung-Hagesteijn et al., 2001; Morfini et al., 2004; Lee et al., 2005; Szatmari et al., 2005; Bertrand et al., 2012). However, the lack of reagents that specifically detect nonphospho-S9 (npS9) GSK3 has limited our ability to directly study dephosphorylation of this N-terminal serine. The activity of GSK3 is augmented by the phosphorylation of tyrosine 216 in GSK3β or tyrosine 279 in GSK3α, (Hughes et al., 1993; Frame and Cohen, 2001), but tyrosine phosphorylation appears to be mostly derived from chaperone-dependent autophosphorylation during (or shortly after) translation and stabilizes the enzymes (Wang et al., 1994a; Cole et al., 2004; Lochhead et al., 2006).

Reagents for specifically assessing changes in the pool of “active” GSK3β/α (i.e., npS9/21) do not exist. Currently, researchers rely on the use of phospho-S9 GSK3β or phospho-S21 GSK3α antibodies for detecting “inactive” enzymes. These approaches only indirectly measure active GSK3 by comparing changes in the ratio of phospho-Ser GSK3 to total GSK3. Moreover, kinase activity assays are available, but the required specificity for GSK3β is not currently possible in lysates. Instead, GSK3β activity assays are performed by immunoprecipitating GSK3β and then measuring activity with GSK3β substrate peptides or proteins (Welsh et al., 1997; Bijur and Jope, 2001; Bowley et al., 2005). Reagents that directly detect the amount of npS9 GSK3β and can be used in GSK3β kinase activity assays would provide obvious advantages to biochemical analyses and allow localization within cells and tissues. Thus, we set out to generate novel monoclonal antibodies against npS9 in GSK3β because of the lack of such reagents, involvement of GSK3β in several processes and the broad interest in GSK3β across many fields.

## Materials and Methods

### Synthetic GSK3 Peptides

The GSK3β immunization peptides were synthesized and Keyhole Limpet hemocyanin (KLH) was conjugated to the N-termini by GenScript (Piscataway, NJ, USA). The following peptides were generated using variations within the first 14 amino acids of human GSK3β (Uniprot ID: P49841), (1) N-term KLH npS9 GSK3β peptide: 1-14 GSK3β (KLH-^1^MSGRPRTT**S**FAESC^14^), (2) arginine enantiomer npS9 GSK3β peptide (KLH-^1^MSG[d-R]PRTT**S**FAESC^14^) and (3) tandem npS9 GSK3β peptide (KLH-^4^RPRTT**S**FAES^13^/^4^RPRTT**S**FAES^13^). These KLH-peptide reagents were >90% pure and stocks at 2 mg/ml were diluted in H_2_O and stored at -20°C until used for immunizations as described below. The following four peptides GSK3β and α screening peptides were used for screening during monoclonal antibody production, (1) npS9 GSK3β (^1^MSGRPRTT**S**FAESCKPVQQPSAFGS^25^), (2) pS9 GSK3β (^1^MSGRPRTT[**pS**]FAESCKPVQQPSAFGS^25^), (3) npS21 GSK3α (^10^GPGGSGRARTS**S**FAEPGGG^28^) and (4) pS21 GSK3α (^10^GPGGSGRARTS[**pS**]FAEPGGG^28^). These peptides were >90% pure and stocks at 1 mg/ml diluted in H_2_O were stored at -20°C until used in assays as indicated below.

### Animal and Human Subjects

Three-month-old BALB/c female mice were used for immunizations. A male C57BL/6J wild-type mouse and male Fisher 344 rat were perfused with 0.9% saline containing heparin for fresh tissue samples. A male Fisher 344 rat was perfused for fixed tissue as described Grabinski et al. (2015). Timed-pregnant female Sprague Dawley rats were used to obtain E18 fetal cortical cells for primary neuron cultures as described Grabinski et al. (2015). All studies involving animals were conducted in compliance with federal, state and institutional guidelines and approved by the Michigan State University Institutional Animal Care and Use Committee.

Fresh frozen tissue from the frontal cortex of an aged, non-demented case (sex: male, age: 75 years, post-mortem interval: 2.5 h) was used for biochemical analyses, and fixed tissue from an aged non-demented case was used (sex: male, age: 80 years, post-mortem interval: 3.25 h) for the immunohistochemical analysis. The Michigan State University Institutional Review Board approved all post-mortem human tissue studies for human subject’s exemption. All of the samples were de-identified and obtained through the Alzheimer’s Disease Research Center at Banner Sun Health Research Institute (Beach et al., 2015).

### Monoclonal Antibody Production

Animals received injections of the N-terminal peptide, enantiomer peptide, tandem repeat peptide, or a mixture of all three peptides (200 μg peptide) every 3 weeks and serum was collected 3 days after the indicated immunizations to determine antibody titer. Once serum titers showed above-background signal at ≥1:51,200 dilution, the animal was boosted again and 3 days later used for the fusion procedure. Hybridoma fusion techniques similar to those described previously were used to create monoclonal antibodies (Binder et al., 1985). Cultures were screened for reactivity against npS9 GSK3β, pS9 GSK3β, pS9 GSK3β and pS21 GSK3β screening peptides by indirect ELISAs 7–10 days after plating. Cultures that were positive (i.e., typically absorbance >1.0) were expanded, screened again and then plated into 96-well (∼1 cell/well for first clone plating). Clones were screened again, and the strongest wells were expanded in medium consisting of RPMI-1640 supplemented with 1x HT (11067-030, Thermo, Waltham, MA, USA), 10% Fetal bovine serum, 5% L-Glutamine, 1% sodium pyruvate, and 1% penicillin-streptomycin. Cells were subcloned following this process at least three times and we require >95% of all wells are positive in ELISAs. The third subclones were subjected to freeze/thaw cycles (Hybridoma Freezing Medium: RPMI-1640, 10% FBS, 5% L-Glutamine, 1% sodium pyruvate, 1% penicillin-streptomycin and 5% DMSO) for a total of three times to establish stability upon liquid nitrogen freezing and retrieval. Antibody isotypes were determined using the IsoStrip Mouse Monoclonal Antibody Isotyping Kit (11493027001, Roche) and mycoplasma testing was performed using the Mycoplasma PCR ELISA kit (11663925910, Roche). Once the clones were verified as clean, stable and positive the antibodies were purified.

Each antibody was produced using CELLine 350 bioreactors (Integra Biosciences, Hudson, NH, USA) according to the manufacturer’s instructions. Culture supernatants were dialyzed overnight in 1.5 M glycine, 3 M NaCl buffer, pH 8.9 (Protein A buffer) using dialysis tubing (PI68100, Thermo). Dialyzed culture supernatant was centrifuged at 12,000 × *g* for 20 min and filtered (5 μm pore filter) and purified using rProtein A Sepharose Fast Flow resin (17-1279-01, GE Healthcare, Pittsburgh, PA, USA). The antibodies were eluted using 0.1 M citric acid starting at pH 6, followed by pH 5, pH 4, and pH 3 (10 ml each). Fractions were collected (5 ml), run on 4–20% Tris-HCl Criterion gels (567–1093, BioRad, Hercules, CA, USA) and stained by Coomassie. Fractions containing IgGs were pooled and concentrated in an Amicon Ultra Centrifugal Unit (UFC90-30-24, Thermo) and dialyzed overnight in antibody storage buffer (10 mM HEPES, 500 mM NaCl, 50% Glycerol). Concentrations were measured using A280 (extinction coefficient of 13.7) and antibodies were adjusted to 1 mg/ml, aliquoted and stored at -80°C.

### Indirect ELISAs

Indirect ELISAs were performed to determine the binding affinity and specificity of each of the antibodies for non phospho and phospho GSK3β and GSK3α peptides as described Kanaan et al. (2011). For the antibody titer ELISAs, 50 μl of the GSK3 screening peptides (without KLH) were diluted to 2 ng/μl in a borate saline solution (100 mM boric acid, 25 mM sodium tetraborate decahydrate, 75 mM NaCl, 250 μM thimerosal) and wells (Corning, #3590) were coated for 1 h. Between all steps, wells were washed with ELISA wash solution (100 mM boric acid, 25 mM sodium tetraborate decahydrate, 75 mM NaCl, 250 μM thimerosal, 0.4% bovine serum albumin and 0.1% tween-20; 200 μl/well). Wells were blocked with 200 μl 5% non-fat dry milk made in ELISA wash solution (blocking buffer) for 1 h. The purified GSK3 antibodies were serially diluted in blocking buffer at a range from 1:400 (2500 ng/ml or 16.7 nM) to 1:819,200 (1.22 ng/ml or 6.7 pM) and incubated for 2 h. Goat anti-mouse HRP conjugated antibody (115-035-003, Jackson ImmunoResearch, West Grove, PA, USA) was added to each well at a dilution of 1:5,000 for 1 h. Reactivity was detected by adding 3,3′,5,5′ tetramethylbenzidine substrate (T0440, Sigma Aldrich, St. Louis, MO, USA) to each well and incubated for approximately 8 min. Reactions were quenched with 50 μl 3.6% H_2_SO_4_ and then the absorbance at 450 nm. Blank wells were used to obtain background absorbance, which was removed from sample signals. For antibody specificity ELISAs, the assays were run as described above with the exception that the plates were coated with a wide range of either npS9 GSK3β, pS GSK3β, npS21 GSK3α, or pS21 GSK3α peptides (0 – 6.4 μg/well). The peptides were detected using 12B2 (2 nM) or 15C2 (1 nM) primary antibodies and signals were detected and analyzed as above. Finally, we confirmed the presence of phosphorylation at S9 in the pS9 GSK3β peptide (well coated with 50 μl at 2 ng/μl) using the phosphoS9 GSK3β primary antibody (1:1,000; 9323, Cell Signaling).

### Cell Culture

Primary neurons from E18 rat cortex were cultured for 8 days as described previously (Grabinski et al., 2015). Human embryonic kidney (HEK) 293T cells (CRL-3216, ATCC) were grown in DMEM (11995-065, Thermo) supplemented with 5% FBS and 1% penicillin/streptomycin (15140-122, Thermo). Human neuroblastoma SH-SY5Y cells (CRL-2266, ATCC) were grown in Ham’s F-12 medium (11765-054, Thermo) mixed with Minimum Essential Medium (11095-080, Thermo) at a ratio of 1:1 and supplemented with 5% FBS and 1% penicillin/streptomycin. Human glioblastoma U373 cells and mouse neuroblastoma Neuro-2A cells were grown in DMEM/Ham’s F12 (10-090-CV, Corning) supplemented with 10% FBS, 2mM L-glutamine (25030-081, Thermo), 1% MEM Non-Essential Amino Acids (11140-050, Thermo), and 1 mM sodium pyruvate (25000CI, Corning). Cells were collected and processed for the specific assays as outlined below.

### siRNA Knockdown

HEK293T cells were plated in a 24-well plate at a density of 60,000 cells/well in 400 μl growth medium without antibiotics (DMEM (11995-065, Thermo) supplemented with 5% FBS) 1 day prior to transfection. HEK293T cells were transfected with either the human GSK3β Stealth primer set (1299001-VHS40279, Thermo), the human GSK3α Stealth primer set (1299001-HSS104518, Thermo), the human GAPDH Stealth control (12935140, Thermo), or the medium GC siRNA negative control (12935300, Thermo) constructs according to manufacturer’s protocol. The GAPDH siRNA controls for expression of a functional siRNA against an unrelated target, and the medium GC siRNA is an additional control for non-specific effects. The siRNA constructs were diluted to 100 pM for GSK3α, 100 pM for GSK3β, 40 nM for GAPDH or 10 nM for medium GC in 50 μl OPTI-MEM (31985-070, Thermo). Lipofectamine 2000 (11668-027, Thermo) was mixed at a ratio of 1–50 μl OPTI-MEM and incubated at room temperature for 15 min. Then, the siRNAs and Lipofectamine 2000 were combined and incubated at room temperature for 15 min. After the incubation, the reagents were added to the cells (100 μl/well) and incubated for 48 h before collecting the cells for Western blotting and immunocytofluorescence as described below.

### 12B2 and 15C2 Antibody Immunoprecipitations

Immunoprecipitations were performed by conjugating either 12B2, 15C2 or a non-immune mouse IgG to NHS Magnetic Sepharose beads according to the manufacturer’s instructions (28944009, GE Healthcare). Magnetic beads were prepared by briefly equilibrating 25 μl of the bead slurry into ice cold 1 mM HCl, then immediately removing equilibration buffer and adding 200 μl of the antibody at 25 ng/μl (5 μg total antibody diluted in phosphate buffered saline: 137 mM, NaCl, 2.68 mM KCl, 10 mM Na_2_HPO_4_, 1.76 mM KH_2_PO_4_, pH 7.4). The antibodies were bound to the beads during a 40 min incubation at room temperature with end over end mixing. Residual NHS active groups were blocked following a series of washes and incubations with two separate reagents. Beads were washed with 500 μl blocking buffer A (50 mM tris-HCl, 1M NaCl, pH 8.0), followed by washing with 500 μl blocking buffer B (50 mM glycine-HCl, 1M NaCl, pH 3.0), followed by incubation in 500 μl blocking buffer A for 15 min with end over end mixing. Another series of washes occurred starting with blocking buffer B, followed by blocking buffer A, and then blocking buffer B. After removing the final blocking buffer B the IgG-bound beads were resuspended in 500 μl TBS and transferred to a new tube. HEK293T cells were collected in lysis buffer (20 mM tris, pH 7.5, 2.5mM DTT, 1% Triton X-100, 300 mM NaCl)), sonicated and spun at 12,000 × *g* for 10 min to remove debris and the supernatant was used for the immunoprecipitations. The beads were incubated with 500 μg total protein of HEK293T lysate with end over end mixing for 1 h at room temperature. Lysate samples prior to incubation with the beads were reserved as the “Input” sample for western blotting. After samples were incubated with beads, the unbound sample was removed and saved for use as the “Post-IP” sample for western blotting. The beads were washed 5x with 500 μl TBS before transferring to a new tube. The final wash was removed and beads were resuspended in 25 μl 2x Laemmli sample buffer. SDS-PAGE and western blotting were done using 50 μg “Input” and “Post-IP” samples, and 12.5 μl of each IP sample was used for the “12B2-IP,” “15C2-IP” and “Ms IgG-IP” samples. The IP experiments were repeated three independent times and western blots (12B2, 15C2, and IgG control) were probed with rabbit anti-GSK3 α/β total antibody (1:2,000, 5676, Cell Signaling) and imaged as described below.

### Western Blotting

Purified npS9 GSK3 antibodies were validated with recombinant GSK3β and α protein and brain lysates from human, mouse and rats (Kanaan et al., 2011, 2012). Recombinant protein samples were prepared by using 300 ng GSK3β alone (His-tagged, PV3365, Thermo, Waltham, MA, USA) or GSK3α alone (GST-tagged, PV6126, Thermo, Waltham, MA, USA), GSK3β or α incubated with alkaline phosphatase (6 U per reaction; EF0651, Fermentas, Waltham, MA, USA) for 4 h at 30°C to create a non-phosphorylated GSK3 sample, and GSK3β or α incubated with 2 μg Akt (14-276, Millipore, Billerica, MA, USA) and ATP (400 μM) for 4 hrs at 30°C to create a phosphorylated Ser GSK3 sample. The samples were brought to a final concentration of 8.33 ng/μl in 2x Laemmli sample buffer at 98° C for 10 min. Human, mouse, and rat frontal cortex homogenates and cell culture lysates (primary neurons were grown for 8 days at 45,000 cells/cm^2^ and cell lines were collected when ∼80–90% confluent) were prepared by sonicating samples in lysis buffer (20 mM Tris, pH 7.5, 2.5 mM DTT, 300 mM NaCl, 1% Triton X-100). Samples were centrifuged at 12,000 × *g* for 10 min at 4° C and supernatants collected. Total protein concentrations were determined using the Bradford protein assay (500-0006, BioRad, Hercules, CA, USA).

Recombinant GSK3 samples were loaded at 50 ng/lane, while the brain lysates and cell culture lysates were loaded at 50 μg/lane and separated using SDS-PAGE on 4–20% Tris-HCl Criterion gels (BioRad). Proteins were transferred onto PVDF membrane (IPFL00010, Millipore, Billerica, MA, USA) and probed with either 12B2 (1:1,000) or 15C2 (1:3,000) and total GSK3 α/β (1:2,000; 5676, Cell Signaling), or with pS9 GSK3β (1:1,000; 9323, Cell Signaling) and Ms total GSK3β (1:2,000 9832, Cell Signaling) antibodies diluted in 5% non-fat dry milk in tris buffered saline (TBS, 0.5 M Tris, 1.5 M sodium chloride, pH 7.4) overnight at 4° C. Brain lysate blots were also probed with anti-GAPDH antibody (1:2,000; 5174, Cell Signaling) for a loading control. The following day the blots were incubated for 1 h at with IRDye 800CW goat anti-mouse IgG (H + L) (1:20,000; 926-32210, LiCor Biosciences, Lincoln, NE, USA) and IRDye 680LT goat anti-rabbit IgG (H + L) (1:20,000; 926-68021, LiCor Biosciences) for 12B2 and 15C2 antibody blots, or IRDye 680LT goat anti-mouse IgG (H + L) (1:20,000; 926-68020, LiCor Biosciences) and IRDye 800CW goat anti-rabbit IgG (H + L) (1:20,000; 925-32211, LiCor Biosciences) for pS9 GSK3β blots. Immunoblots from the Akt inhibitor and protein phosphatase inhibitor experiments (see below) were probed with total Akt (1:2,000; 2920, Cell Signaling) and either phospho-Thr308 Akt (1:1000; 13038, Cell Signaling) or phospho-Ser473 Akt (1:2,000; 4060, Cell Signaling) antibodies, and detected with IRDye 680LT goat anti-mouse IgG (H + L) and IRDye 800CW goat anti-rabbit IgG (H + L) as above. Then blots were imaged using a Licor Odyssey system. Band signal intensities were quantified using ImageStudio software (v5.2.5, LiCor Biosciences). The signals for 12B2, 15C2 and the pS9 GSK3β antibodies were normalized to total GSK3 signal in the recombinant GSK3 blots.

### Cell Culture Immunocytofluorescence

Primary neurons from E18 rat cortex, HEK293T and SH-SY5Y cells were plated at 30,000 cells/well in 8-well chamber slides coated with poly-D-lysine (to enhance cell adherence). Neurons were grown for 8 days, while HEK293T and SH-SY5Y cells were grown for 2 days before being fixed for 20 min at room temperature using 4% paraformaldehyde in cytoskeletal buffer (10 mM MES, 138 mM KCl, 3 mM MgCl_2_, 4 mM EGTA, pH 6.1). Fixed cells were rinsed 3x in TBS for 5 min each and then blocked and permeabilized with 5% goat serum/1% BSA/0.2% Triton-X for 1 hr at room temperature. Cells were stained with one of the GSK3 primary antibodies diluted in 2% goat serum and incubated overnight at 4° C (12B2 – 1:100; 15C2 – 1:100; total GSK3 α/β – 1:200). Cells were incubated in AlexaFluor goat anti-mouse IgG1 488 (A21121, Thermo) for 12B2 or 15C2 and AlexaFluor goat anti-rabbit 568 (A11036, Thermo) for total GSK3 β/α (all diluted 1:500 in 2% goat serum). DAPI counterstain (0.5 μg/ml, D1306, Thermo) was added to the first of four TBS rinses. Control stains were performed where the primary antibodies were omitted to confirm that each secondary label was specific to the appropriate primary antibody (**Supplementary Figure S3A**). Image z-stacks (0.5 μm step size) were taken using a Nikon A1+ laser scanning confocal microscope system equipped with 488, 561, and 640 solid-state lasers, Nikon Elements AR software, and the images (maximum intensity projections) were prepared for publication using Adobe Photoshop and Illustrator.

### Tissue Immunohistochemistry

Immunohistochemistry was performed on human control (temporal lobe) and rat tissue sections (40 μm thick) following established protocols (Kanaan et al., 2016). The tissue was incubated with purified GSK3 antibodies (12B2 – 1:500 or 15C2 – 1:1,000) overnight at 4° C, followed by goat anti-mouse biotinylated secondary antibody at 1:500 (115-065-166, Jackson Immuno Research) and then ABC Elite solution (according to the manufacturer’s instructions; PK-6100, Vector Labs, Burlingame, CA, USA). The tissue was developed using 3,3′-diaminobenzidine (D5637, Sigma) at 0.5 mg/ml in TBS-Tx with 0.003% H_2_O_2_ for 8 min. Control sections that were stained following the same procedure, but without the primary antibodies were performed (**Supplementary Figure S3B**). Images were acquired as z-stacks (0.9 μm step size) with a Nikon Eclipse 90i microscope, a Nikon DS-Ri1 camera, and Nikon Elements AR software (Nikon Instruments Inc., Melville, NY, USA), and the images (displayed using the extended depth of focus function) were prepared for publication using Adobe Photoshop and Illustrator.

### GSK3β Kinase Activity Assays

The GSK3β Kinase Enzyme System kit (V1991, Promega) was used to generate a standard curve of GSK3β activity per the manufacturer’s instructions. The active GSK3β enzyme (G09-10G, lot P1578-8, Signal Chem) was brought to final concentrations of 12, 9.6, 7.2, 4.8, and 2.4 ng/μl in 25 μl reaction mixture containing 100 μM ATP (V915A, Promega) in reaction buffer (40 mM Tris-HCl, pH 7.4, 20 mM MgCl_2_ and 0.1 mg/ml bovine serum albumin; supplied as a 5x stock). The reactions were incubated at 30° C for 15 min. The reactions were terminated and remaining ATP depleted by adding 25 μl ADP-Glo^TM^ reagent (V912A, Promega) made according to the manufacturer’s instructions. After 40 min incubation at room temperature, 50 μl of the kinase detection reagent (V913A and V914A, Promega, made according to manufacturer’s instructions) was added to the reaction and incubated for 30 min at room temperature. All samples were run in triplicate. Then the luminescence was measured using a GloMax Muli-Detection plate reader (E7031, Promega). The relative luminescence units are directly proportional to the amount of ATP used during the kinase reaction.

### Nonphospho-S9 GSK3β Western Blotting Curve

The 12B2 and 15C2 GSK3β antibodies were tested using samples comprised of known amounts of npS9 GSK3β and pS9 GSK3β. The npS9 GSK3β was obtained by incubating GSK3β (1 μg, Thermo, PV3365) with alkaline phosphatase (15 U, Thermo, EF0654) for 5 h at 30° C. The pS9 GSK3β was generated by incubating GSK3β (1 μg) with Akt1 (1 μg, P2999, Thermo) and ATP (1 mM, N0440S, New England BioLabs) in kinase buffer (20 mM Tris-HCl, pH 7.5, 10 mM MgCl_2_, 5 mM DTT) for 5 h at 30°C. Akt1 is a kinase known to phosphorylate S9 in GSK3β (Cross et al., 1994, 1995). The following seven samples were generated that matched the levels of npS9 GSK3β used in the kinase activity assay curve above: (1) 0% npS9 GSK3β (300 ng/lane pS9 GSK3β), (2) 10% npS9 GSK3β (30 ng + 270 ng/lane), (3) 20% npS9 GSK3β (60 ng + 240 ng pS9 GSK3β), (4) 40% npS9 GSK3β (120 ng + 180 ng pS9 GSK3β), (5) 60% npS9 GSK3β (180 ng + 120 ng pS9 GSK3β), (6) 80% npS9 GSK3β (240 ng + 60 ng pS9 GSK3β), and (7) 100% npS9 GSK3β (300 ng + 0 ng pS9 GSK3β). The samples were added to Laemmli sample buffer, loaded at a total of 300 ng/lane, and separated on a 7.5% TGX precast Criterion gel for SDS-PAGE and subsequently processed for western blotting using 12B2 or 15C2 antibody and total GSK3β α/β as described above.

### HEK293T Cell Culture Calyculin A Treatment

HEK293T cells were grown for 48 hrs and then treated with 10 nM calyculin A (9902S, Cell Signaling Technology, Beverly, MA, USA), a potent protein phosphatase inhibitor, for 30 min to induce phosphorylation of S9 GSK3β prior to being collected in lysis buffer (as above). Lysates were sonicated and centrifuged at 12,000 × *g* for 10 min, the supernatants were collected the Bradford Protein Assay was used to determine total protein concentrations before being used in sandwich ELISA and GSK3β activity assays.

The same lysates were used in a sandwich ELISA as described previously (Kanaan et al., 2016). Briefly, all of these assays were performed by coating wells in 96 well plates (Corning, #3590) with 12B2 antibody (250 ng/well) as the capture antibody, wells were blocked with 5% non-fat dried milk (1 h), then incubated with the samples (see below), then incubated with rabbit anti-total GSK3 α/β antibody (0.5 μg/ml, 1.5 h, same antibody as above), and then incubated with goat anti-rabbit antibody conjugated to horseradish peroxidase (Vector Labs, PI-1000; (0.2 μg/ml; 1.5 h). Signal was detected with TMB for 25 min and then the reaction was stopped using 3.6% H_2_SO_4_. The absorbance at 450 nm was measured and blank wells were used to obtain background absorbance, which was removed from sample signals. The following sandwich ELISAs sample sets were assayed. A recombinant npS9 GSK3β protein standard was run to determine the amount of bound npS9 GSK3β in the experimental lysate samples. Briefly, 2 μg of recombinant GSK3β (G09-10G, Signal Chem) was incubated with 60 U alkaline phosphatase (EF0651, Fermentas) at 30° C for 2 h to create the npS9 GSK3β protein standard. Then 300-0.29 ng/well npS9 GSK3β was used to create the standard curve (triplicate samples per concentration). The absorbance values were log_10_ transformed and fit to a sigmoidal curve (*r*^2^= 0.999) and this was used to interpolate the protein amounts in the experimental HEK cell lysate samples. A HEK293T lysate standard curve also was run to establish that the assay produces linear detection of npS9 GSK3β amounts from lysate samples. The HEK293T lysate samples were applied at 7.5, 15, 30, 60, and 120 μg. Finally, HEK293T lysates from control and calyculin A treated cells were incubated in the sandwich ELISAs (60 μg protein). For these experimental samples, the absorbance values were used to interpolate unknown values of npS9 GSK3β levels from the recombinant GSK3β standard curve assay (from above) and the interpolated values were used for comparisons between untreated control cells and calyculin A treated cells.

The same lysates were also used to measure GSK3β kinase activity using an assay that combined the sandwich ELISA approach with the GSK3β kinase activity assay described above. Wells were coated with 12B2 (250 ng/well, diluted in borate saline), blocked and then the samples were applied. Three sample sets were tested, (1) a GSK3β enzyme standard (300 – 9.4 ng, diluted in TBS, G09-10G, Signal Chem), (2) control HEK cell lysates and calyculin A (10 nM) treated HEK cell lysates, and (3) both HEK cell lysates with a TCS-2002 (100 μM), a GSK3β-specific inhibitor (Saitoh et al., 2009). The GSK3β standard was used to ensure the experimental sample signals were within the linear range of the assay. The control and calyculin A treated lysates were used to confirm whether the changes in npS9 GSK3β levels seen in western blotting and sandwich ELISAs were related to an actual reduction in GSK3β kinase activity. The TCS-2002 inhibitor was used to confirm that the signals were derived from GSK3β. After sample incubation, the wells were rinsed and incubated in 50 μl/well GSK3β activity reaction buffer [100 μM ATP (V915A, Promega), 40 mM Tris-HCl, pH 7.4, 20 mM MgCl_2_ and 0.1 mg/ml bovine serum albumin] for 1 h at 30°C. Then, the kinase reaction was stopped with 50 μl/well ADP-Glo^TM^ reagent (V912A, Promega) made according to the manufacturer’s instructions. After 40 min incubation at room temperature, 100 μl of the kinase detection reagent (V913A and V914A, Promega, made according to manufacturer’s instructions) was added to the reaction and incubated for 30 min at room temperature. Then the luminescence was measured using a GloMax Muli-Detection plate reader. The relative luminescence units are directly proportional to the amount of ATP used during the kinase reaction.

### Akt Inhibitor and Protein Phosphatase Treatments in HEK293T Cell Cultures

HEK293T cells were grown for 48 hrs and then treated with either nothing (control), an Akt-specific inhibitor (AZD-5363, 1 μM, 15406, Cayman Chemical) (Davies et al., 2012; Li et al., 2013), a protein phosphatase inhibitor (calyculin A, 10 nM, 9902S, Cell Signaling Technology) or both AZD-5363 and calyculin A. In the dual treatment cultures, cells were first treated with AZD-5363 for 1 h to inhibit the action of Akt on GSK3 (i.e., phosphorylation at S9/21), and then calyculin A was applied for 30 min to establish whether protein phosphatases dephosphorylate S9/21 independent of the Akt pathway (protein phosphatases can increase Akt activity via causing accumulation of phospho-Akt or they can directly dephosphorylate GSK3). Lysates were sonicated and centrifuged at 12,000 × *g* for 10 min, the supernatants were collected for western blotting analysis as above. The Bradford Protein Assay was used to determine total protein concentrations.

### Statistics and Data Analysis

All curve fitting and statistical analyses were performed using Prism 6.0 (GraphPad Software, Inc.). The sandwich ELISA data from the treatment of HEK293T cells with calyculin A were compared using unpaired *t*-test. The western blotting Akt inhibitor and protein phosphatase inhibitor experiment data were compared using a one-way ANOVA, and *post hoc* comparisons were made using the Holm-Sidak test. Immunoblotting signal for 12B2 and 15C2 were correlated to GSK3β enzyme activity using a Pearson’s *r* correlation analysis. The GSK3β kinase activity assay data were compared using a two-way ANOVA with calyculin treatment and TCS-2002 treatment as the two factors, and *post hoc* comparisons were made using the Holm-Sidak test. All tests were two-tailed and significance set at *p* < 0.05.

## Results

### Immunization with npS9 GKS3β Peptides

Mouse N00 was immunized with the N-terminal KLH npS9 GSK3β peptide and animals T10 and E10 were immunized with a mixture of 3 peptides (N-term KLH, arginine enantiomer and the tandem npS9 GSK3β peptides). All of the animals exhibited strong titers against npS9 GSK3β and no detection of the pS9 GSK3β peptide (**Supplementary Figures S1A,B**). Animal T10 had the highest antibody titer after the third immunization boost (A_450_= 0.061 at 1:25,600; **Supplementary Figure S1A**) and was used for the first fusion. Animal N00 had the highest titer after the 6th immunization boost (A_450_= 0.374 at 1:25,600; **Supplementary Figure S1B**) and was used for a second fusion. Hybridoma fusion cultures were screened for reactivity with the npS9 GSK3β, pS9 GSK3β and npS21 GSK3α peptides to determine their specificity prior to subcloning (**Supplementary Figures S1C,D**). The 12B2 cultures showed stronger reactivity for npS9 GSK3β over npS21 GSK3α, and did not react with pS9 GSK3β. The 15C2 culture showed slightly stronger reactivity with npS21 GSK3α over npS9 GSK3β, and did not react with pS9 GSK3β. These cultures were subcloned three times, and with each subcloning step the clones with the strongest reactivity were continued forward (screened against the above peptides in indirect ELISAs) and selected for production and purification. Here, we characterized clone 12B2 (npS9 β-specific) and clone 15C2 (npS β/α-specific), both of which produce IgG1 isotype antibodies.

### 12B2 and 15C2 Antibody Specificity

**FIGURE 1:**
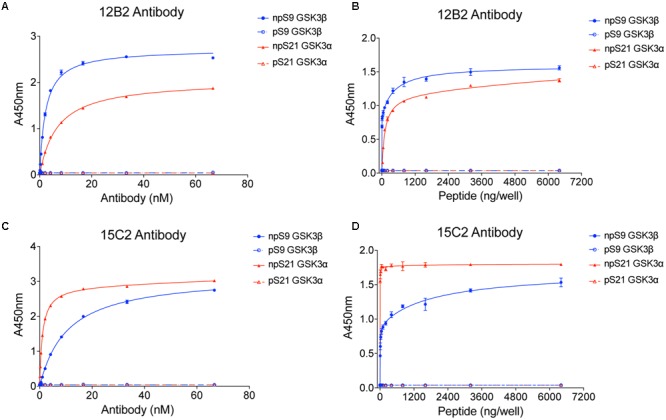
**12B2 and 15C2 are specific for nonphospho-Ser GSK3β/α peptides.** Each antibody was screened in indirect ELISA titers against npS9 GSK3β, pS9 GSK3β, npS21 GSK3α and pS21 GSK3α peptides (*n* = 3 independent experiments). **(A)** 12B2 showed strong reactivity for npS9 GSK3β compared to npS21 GSK3α peptides and did not react with pS9 or pS21 GSK3 peptides (EC_50_ values: npS9 = 2.1 nM; pS9 = indeterminate (id); npS21 = 6.4 nM; pS21 = id). **(B)** To further confirm the specificity of 12B2, ELISAs were performed by coating wells with a wide range of peptide amounts (0 – 6.4 μg peptide/well). 12B2 showed strong reactivity with the npS GSK3 peptides (β > α), but did not react with pS GSK3 peptides. **(C)** 15C2 showed stronger reactivity for npS21 GSK3α compared to npS9 GSK3β and did not react with pS9 or pS21 GSK3 peptides (EC_50_ values: npS9 = 2.4 nM; pS9 = id; npS21 = 277 pM; pS21 = id). **(D)** To further confirm the specificity of 15C2, ELISAs were performed by coating wells with a wide range of peptide amounts (0 – 6.4 μg peptide/well). 15C2 showed strong reactivity with the npS GSK3 peptides, but did not react with pS GSK peptides. It is noteworthy that synthetic peptides provide a homogeneous source of modified peptides, and thus, are ideal for challenging the specificity of the antibodies against nonphospho-Ser and phospho-Ser residues in GSK3.

Once purified, we titered the antibodies using npS9 GSK3β, pS9 GSK3β, npS21 GSK3α and pS21 GSK3α peptides in indirect ELISAs. Clone 12B2 showed a higher affinity for npS9 GSK3β (2.3 nM) compared to npS21 GSK3α (6.8 nM; **Figure 1A**). Clone 15C2 had higher affinity for npS21 GSK3α (1.2 pM) compared to npS9 GSK3β (10.6 nM; **Figure 1C**). Neither of the antibodies reacted with pS9 GSK3β or pS21 GSK3α peptides above background levels demonstrating their specificity for npS GSK3 across a broad range of antibody dilutions (starting as high as 67 nM or 1:100 dilution; **Figures 1A,C**). Next, we further tested the limitations of 12B2 and 15C2 specificity across a broad range of peptide concentrations (0–6.4 μg/well). There was strong reactivity with the npS peptides, but again, no signals above background for the phospho-Ser peptides confirming the specificity even at excessively high levels of purified synthetic phospho-Ser peptides (i.e., 6.4 μg/well; **Figures 1B,D**). It is noteworthy, that synthetic peptides provide the most homogenous source of either phosphorylated or non-phosphorylated antigens. To confirm that the synthetic pS9 GSK3β peptide contained a phosphorylated-Ser residue we also performed an ELISA using the pS GSK3β-specific antibody, and as expected, there was a strong signal demonstrating the peptide contained phosphoS9 residues (**Supplementary Figure S1E**).

**FIGURE 2:**
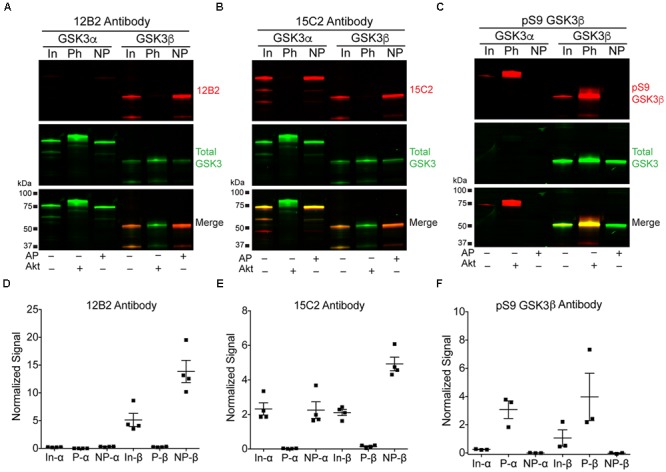
**12B2 is specific for nonphospho-S9 recombinant GSK3β, and 15C2 is specific for nonphospho-S9/21 recombinant GSK3β/α. (A–C)** Western blots of recombinant GSK3β and α alone (input, In), phosphorylated S9 GSK3β and S21 GSK3α proteins (Ph; i.e., Akt1 treated) and dephosphorylated S9 GSK3β and S21 GSK3α proteins (NP; i.e., alkaline phosphatase treated). The GSK3α is GST-tagged and GSK3β is his-tagged, and in each blot the 12B2 **(A)**, 15C2 **(B)** or pS9 GSK3β **(C)** antibodies are red, while total GSK3α/β antibody is green. **(D)** Quantitation of the 12B2 blots shows strong reactivity with npS9 GSK3β, and none with npS21 GSK3α, pS9 GSK3β or pS21 GSK3α. **(E)** Quantitation of the 15C2 blots shows strong reactivity with npS9 GSK3β and npS21 GSK3α, but none with pS9 GSK3β or pS21 GSK3α. **(F)** Quantitation of the pS9 GSK3 blots confirm that the Akt1 treatment produced robust phosphorylation of S9 in GSK3β (and S21 in α) and that the alkaline phosphatase treatment removed phosphorylation of S9 in GSK3β (and S21 in α). Each experiment was repeated three-four independent times and all samples were loaded at 50 ng GSK3/lane. The data are normalized to total GSK3 signal.

Three recombinant GSK3β (his-tagged) and GSK3α (GST-tagged) protein samples were generated: (1) GSK3β or α alone, (2) GSK3s incubated with alkaline phosphatase to dephosphorylate S9 in GSK3β or S21 in GSK3α, or (3) GSK3s incubated with Akt1 to phosphorylate S9 in GSK3β or S21 in GSK3α. These samples were used to establish the specificity of the 12B2 and 15C2 with full-length GSK3 proteins. Blots were probed with each antibody and a rabbit anti-total GSK3α/β antibody (**Figures 2A,B**), or a rabbit anti-pS9 GSK3β antibody and a mouse anti-total GSK3β antibody (**Figure 2C**). The 12B2 antibody showed strong specificity for npS9 GSK3β and did not react with pS9 GSK3β, npS21 GSKα or pS21 GSK3α proteins (**Figure 2D**). The 15C2 antibody showed a weak preference for npS9 GSK3β over npS21 GSK3α and did not react with pS9 GSK3β or pS21 GSK3α (**Figure 2E**). Phosphorylation of S9/S21 was confirmed using the pS9 GSK3β antibody (**Figure 2F**).

**FIGURE 3:**
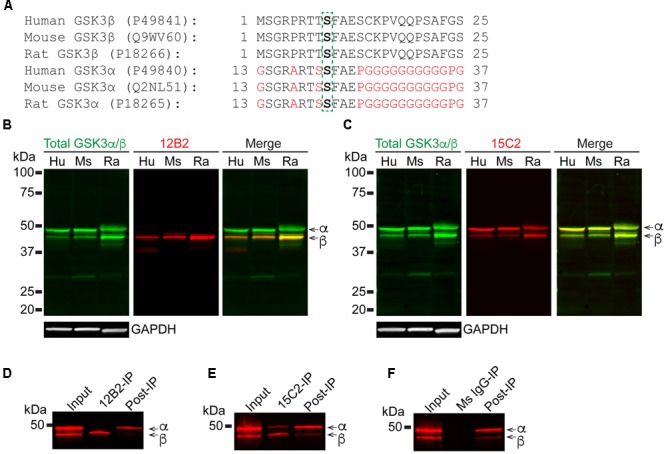
**12B2 and 15C2 are specific for nonphospho-S GSK3 in brain lysates of human, mouse, and rat, and both effectively immunoprecipitate GSK3 from cell lysates. (A)** Protein sequence alignments for GSK3β (amino acids 1–25) and GSK3α (amino acids 13–37) from human, mouse and rat (Uniprot IDs in parentheses). **(B,C)** Blots of lysates from human, mouse and rat cortical tissue and GAPDH was used as a loading control (40 μg/lane total protein loaded; experiment repeated three times). **(B)** 12B2 (red) specifically labeled GSK3β, not GSK3α, in lysates and total GSK3α/β (green) was used to identify both isoforms. **(C)** 15C2 (red) labeled both GSK3β and GSK3α in lysates and total GSK3α/β (green) was used to identify both isoforms. **(D–F)** The 12B2 **(D)**, 15C2 **(E)**, or control mouse IgG (**F**, Ms IgG) were used to immunoprecipitate GSK3 enzymes from HEK293T cell lysates. The starting lysate (Input) was incubated with magnetic beads coated with 12B2 **(D)**, 15C2 **(E)**, or Ms IgG control **(F)** antibodies. 12B2 pulled down only GSK3β (12B2-IP), 15C2 pulled down both GSK3α and β (15C2-IP) and Ms IgG did not pull down GSK3α or β (MsIgG-IP). The post-IP lysates were also run for comparisons to the input samples. These experiments were performed three independent times.

Next, we assessed the specificity and species cross reactivity of 12B2 and 15C2 using human, mouse and rat brain lysates since GSK3β is 100% conserved and there is a notable similarity in GSK3α (**Figure 3A**). Total GSK3α/β antibody detected both forms in humans, mice and rats (**Figures 3B,C**). The 12B2 antibody was specific for npS9 GSK3β and did not react with npS21 GSK3α protein in human, mouse and rat lysates (**Figure 3B**). In contrast, 15C2 detected both npS GSK3 isoforms in human, mouse and rat lysates (**Figure 3C**). Cell lysates from HEK293T (human), SH-SY5Y neuroblastoma cells (human), U373 glioblastoma cells (human), primary neurons (rat), and Neuro-2a neuroblastoma cells (mouse) were used to validate the utility of 12B2 and 15C2 across multiple cell types. In all cell types, 12B2 specifically labeled npS9 GSK3β (**Supplementary Figure S2A**) and 15C2 labeled both npS21 GSK3α and npS9 GSK3β (**Supplementary Figure S2B**), but there were varying degrees of each isoform in the different cell types. We also used HEK293T cell lysates to establish the effectiveness of 12B2 and 15C2 in lysate immunoprecipitations. The 12B2 antibody effectively immunoprecipitated GSK3β only (**Figure 3D**), while the 15C2 antibody pulled down both GSK3α and β (**Figure 3E**). The non-immune mouse IgG control did not immunoprecipitate GSK3α or β (**Figure 3F**).

**FIGURE 4:**
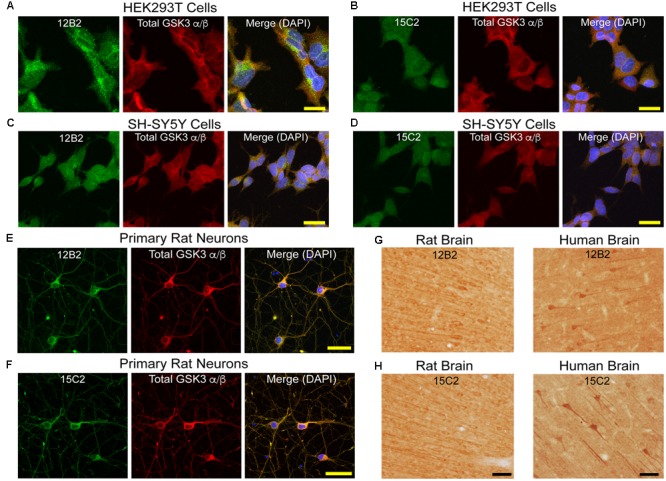
**GSK3β antibody immunostaining in cultured cells and tissue sections. (A,B)** HEK293T cells stained with 12B2 (**A**, green) and 15C2 (**B**, green). (**C,D**) Undifferentiated SH-SY5Y cells stained with 12B2 (**C**, green) and 15C2 (**D**, green). **(E,F)** Rat primary cortical neurons (E18) stained with 12B2 (**E**, green) and 15C2 (**F**, green). In **A-F**, all cells were also stained with total GSK3α/β (red) and DAPI (blue in merged image). The pattern of staining with 12B2 and 15C2 was punctate staining throughout the cells, and 12B2 produced stronger signal than 15C2 in each cell type. Scale bars = 25 μm. **(G,H)** Brain sections in rat (left, retrosplenial cortex displayed) and human (right, temporal cortex) stained with 12B2 **(G)** or 15C2 **(H)**. In general, both antibodies produced clear somatodendritic and parenchymal staining in human and rat brain sections. Scale bars = 50 μm.

Based on the collective results from the assays above, 12B2 is a npS9 GSK3β-specific antibody and 15C2 is a npS9/21 GSK3β/α-specific antibody. Next, we further characterized their reactivity in cell and tissue immunostaining procedures. In general, immunofluorescence for 12B2 produced stronger staining compared to 15C2 (**Figure 4**). The pattern of staining produced by 12B2 was mostly punctate, and this was consistent in HEK cells (**Figure 4A**), SH-SY5Y cells (**Figure 4C**) and E18 rat cortical neurons (**Figure 4E**). The 15C2 antibody produced similar patterns of staining, but the overall signal was not as robust as 12B2 in HEK cells (**Figure 4B**), SH-SY5Y cells (**Figure 4D**) and primary neurons (**Figure 4F**). Primary delete stains confirmed that the pattern of staining was dependent upon the 12B2 and 15C2 antibodies (**Supplementary Figure S3A**).

Brain tissue sections from rat and human brains were processed for IHC. Both 12B2 (**Figure 4G**) and 15C2 (**Figure 4H**) produced reactivity in the somata and processes of neurons, as well as the parenchyma in the rat (retrosplenial cortex depicted) and human brain (temporal lobe depicted). Primary delete sections confirmed the signal was due to the primary antibodies, and not non-specific reactivity from other components of the staining procedure (**Supplementary Figure S3B**).

**FIGURE 5:**
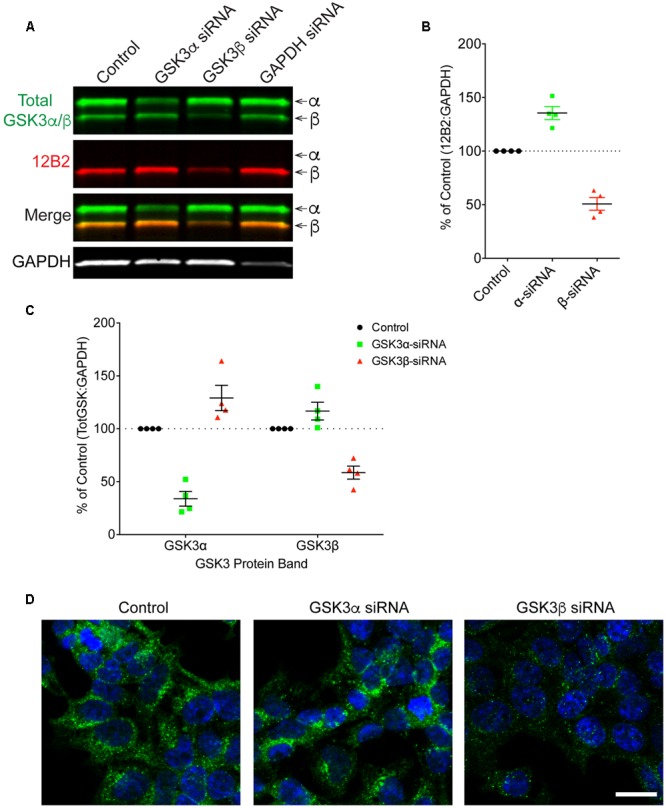
**siRNA knockdown of GSK3α and GSK3β demonstrate specificity of the 12B2 antibody. (A)** HEK293T cells were treated with control, GSK3α, GSK3β or GAPDH siRNAs and probed with 12B2 (red) and total GSK3β/α (green) antibodies. **(B)** Quantitation of 12B2 signal shows that GSK3β siRNA caused a reduction of 50% for GSK3β when compared to control cells, while GSK3α siRNA caused an increase in GSK3β (+35%). **(C)** Quantitation of total GSK3α/β antibody signal shows that GSK3α siRNA caused a loss of 66% for GSK3α and an increase in GSK3β (+29%) when compared to controls. Quantitation of total GSK3α/β antibody signal shows that GSK3β siRNA caused a loss of 41% for GSK3β and an increase in the GSK3α (+17%) when compared to control cells. All immunoblotting data are normalized to GAPDH signal and expressed as percent of the control group to illustrate the siRNA-mediated changes in signal. **(D)** Immunocytofluorescence of HEK293T cells confirms the reduction in 12B2 detection of npS9 GSK3β, which produces a punctate staining pattern, in GSK3β siRNA treated cells compared to control and GSK3α siRNA treated cells. Scale bars = 20 μm. Four independent experiments were performed. GAPDH siRNA quantitation is provided in **Supplementary Figure S4**.

**FIGURE 6:**
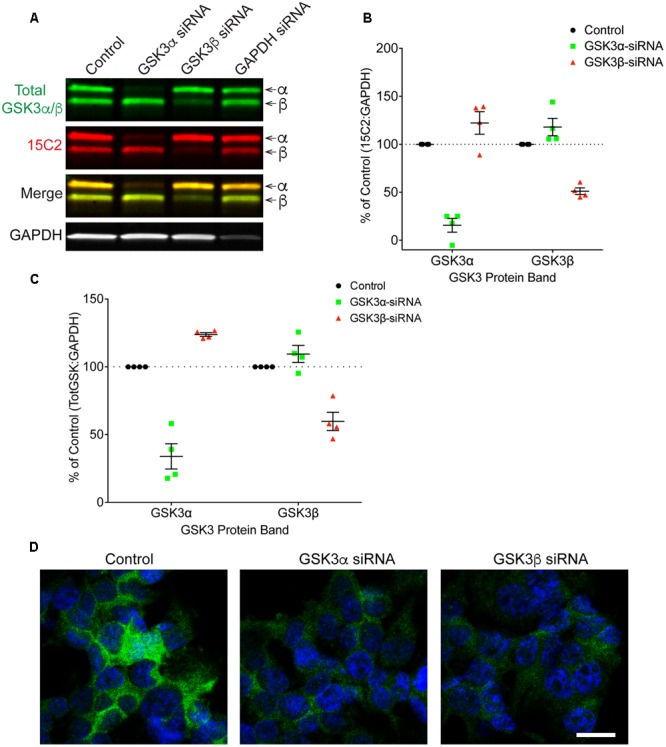
**siRNA knockdown of GSK3α and GSK3β demonstrate specificity of the 15C2 antibody. (A)** HEK293T cells were treated with control, GSK3α, GSK3β or GAPDH siRNAs and probed with 15C2 (red) and total GSK3β/α (green) antibodies. **(B)** Quantitation of 15C2 signal shows that GSK3α siRNA caused a loss of 84% for GSK3α and an increase in GSK3β (+22%) when compared to control cells. Quantitation of 15C2 shows that GSK3β siRNA caused a loss of 49% for GSK3β and an increase in GSK3α (+18%) when compared to control. **(C)** Quantitation of total GSK3α/β antibody signal shows that GSK3α siRNA caused a loss of 66% in GSK3α and an increase in GSK3β (+24%) when compared to controls. Quantitation of total GSK3α/β antibody signal shows that GSK3β siRNA caused a loss of 40% for the GSK3β and an increase in GSK3α (+9%) when compared to control cells. All immunoblotting data are normalized to GAPDH signal and expressed as percent of the control group to illustrate the siRNA-mediated changes in signal. **(D)** Immunocytofluorescence of HEK293T cells confirms the reduction in 15C2 detection of npS21 GSK3α or npS9 GSK3β when treated with GSK3α siRNA or GSK3β siRNA, respectively. Scale bars = 20 μm. Four independent experiments were performed. GAPDH siRNA quantitation is provided in **Supplementary Figure S4**.

Finally, HEK293T cells were treated with siRNAs to further confirm the specificity of 12B2 and 15C2 antibodies. Treatment of cells with GSK3β siRNA reduced 12B2 signal (-50%) in the GSK3β band, while GSK3α siRNA caused an increase in 12B2 signal (+35%) in the GSK3β band compared to control (**Figures 5A,B**). Quantitation of total GSK3α/β antibody signal showed that GSK3α siRNA reduced GSK3α levels (-66%) and increased GSK3β (+29%), while GSK3β siRNA reduced GSK3β (-41%) and increased GSK3α (+17%) when compared to control cells (**Figure 5C**). The reduction in 12B2 signal with GSK3β siRNA was apparent in the reduced signal with immunocytochemistry (**Figure 5D**). Treatment of cells with GSK3α siRNA reduced only the α band (-84%) detected by 15C2, and treatment with GSK3β siRNA reduced only the β band (-49%) detected by 15C2 compared to control (**Figures 6A,B**). With GSK3α siRNA the GSK3β band detected by 15C2 was increased (+22%), and with GSK3β siRNA the GSK3α band detected by 15C2 was increased (+18%) compared to control. Quantitation of total GSK3α/β antibody signal showed that GSK3α siRNA reduced GSK3α levels (-66%) and increased GSK3β (+24%), while GSK3β siRNA reduced GSK3β (-40%) and increased GSK3α (+9%) when compared to control cells (**Figure 6C**). The reduction in GSK3α and GSK3β staining with 15C2 was apparent in the reduced signals with immunocytochemistry (**Figure 6D**). The level of GAPDH knockdown was -82% (**Supplementary Figure S4A**), and GSK3α and GSK3β levels were reduced minimally by ∼17% with GAPDH siRNA treatment (**Supplementary Figure S4B**).

### GSK3β Antibody Reactivity Correlates with GSK3β Enzyme Activity

**FIGURE 7:**
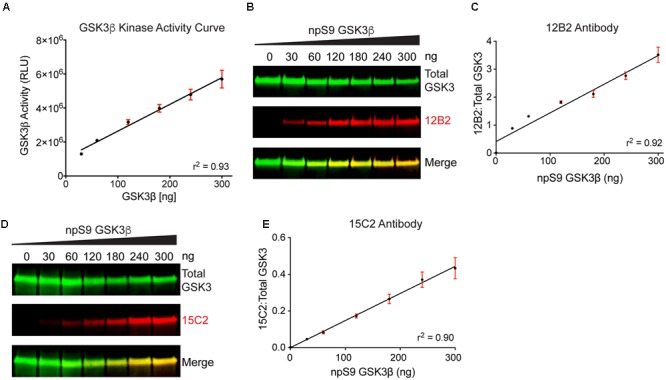
**Detection of recombinant npS9 GSK3β with 12B2 and 15C2 antibodies is linear and correlates with kinase activity. (A)** The level of GSK3β kinase activity with 30, 60, 120, 180, 240, and 300 ng of npS9 GSK3β (“active”) was measured using an *in vitro* GSK3β kinase activity assay and there was a linear increase in kinase activity with increasing amounts of GSK3β (*r*^2^ = 0.93). Three independent experiments were performed. **(B)** For western blotting, recombinant GSK3β was incubated with alkaline phosphatase to generate nonphosphoS9 GSK3β or incubated with Akt1 to generate phosphoS9 GSK3β, and then 0, 30, 60, 120, 180, 240, or 300 ng of npS9 GSK3β was mixed with 300, 240, 180, 120, 60, or 0 ng of pS9 GSK3β to bring the total protein content to 300 ng/lane. The blot was probed with 12B2 (red) and total GSK3α/β antibodies (green). **(C)** Quantitation of signal from 12B2 shows a linear increase in reactivity with increasing npS9 GSK3β amount (*r*^2^ = 0.92). **(D)** The same samples were probed with 15C2 (red) and total GSK3α/β antibodies (green). **(E)** Quantitation of signal from 15C2 shows a linear increase in reactivity with increasing npS9 GSK3β amount (*r*^2^ = 0.90). It is notable that both 12B2 and 15C2 signals also showed a direct correlation with GSK3β activity levels (12B2: *r* = 0.99, *p* = 0.0002; 15C2: *r* = 0.99, *p* < 0.0001). Four independent experiments were performed.

We used recombinant GSK3β proteins for our initial demonstration that 12B2 and 15C2 reactivity reflects GSK3β enzyme activity (**Figures 7A–E**). First, the level of GSK3β activity was assessed using an *in vitro* GSK3β kinase activity assay that uses luminescence to detect the amount of ADP (i.e., ATP used) in a reaction mixture. The assay showed a positive linear dose-response (*r*^2^= 0.93, slope = 15727 ± 1080, *p* < 0.0001) with increasing npS9 GSK3β (30 – 300 ng) (**Figure 7A**). Next, we phosphorylated S9 by incubating GSK3β with Akt1 or dephosphorylated S9 by incubating GSK3β with phosphatase and then brought all samples to the same amount of total GSK3β in each lane (300 ng total/lane containing 0, 10, 20, 40, 60, 80, or 100% npS9 GSK3β). Blots probed with 12B2 (**Figures 7B,C**) and 15C2 (**Figures 7D,E**) showed positive linear reactivity (12B2: *r*^2^= 0.92, slope = 0.0102 ± 0.0006, *p* < 0.0001; 15C2: *r*^2^= 0.90, slope = 0.001478 ± 0.0001, *p* < 0.0001) with increasing npS9 GSK3β. Notably, 12B2 and 15C2 did not show reactivity in the 0% npS9 GSK3β (containing 300 ng phospho-S9 GSK3β) samples confirming the specificity for nonphospho-S9 GSK3 protein. Finally, we correlated 12B2 or 15C2 blotting signal with GSK3β kinase activity levels. Both 12B2 (*r* = 0.99, *p* = 0.0002) and 15C2 (*r* = 0.99; *p* < 0.0001) showed a strong positive correlation with GSK3β activity assay as determined by the luminescence activity assay.

### Protein Phosphatase Inhibition Decreases npS9 GSK3β Levels and GSK3β Enzyme Activity in Cells

To provide a proof-of-principle demonstration of how these new reagents can be used to gain biological insights we focused on the 12B2 antibody because of its specificity for the GSK3β isoform. We used 12B2 to examine the regulation of GSK3β by protein phosphatases in cells. We treated HEK cells for 30 min with 10 nM calyculin A, a potent protein phosphatase inhibitor (Ishihara et al., 1989; Resjo et al., 1999), which increases S9 phosphorylation in GSK3β (Morfini et al., 2004; Kim et al., 2009; Xiao et al., 2010). The cell lysates were analyzed in sandwich ELISAs, GSK3β kinase activity assays, immunofluorescence, and western blot.

**FIGURE 8:**
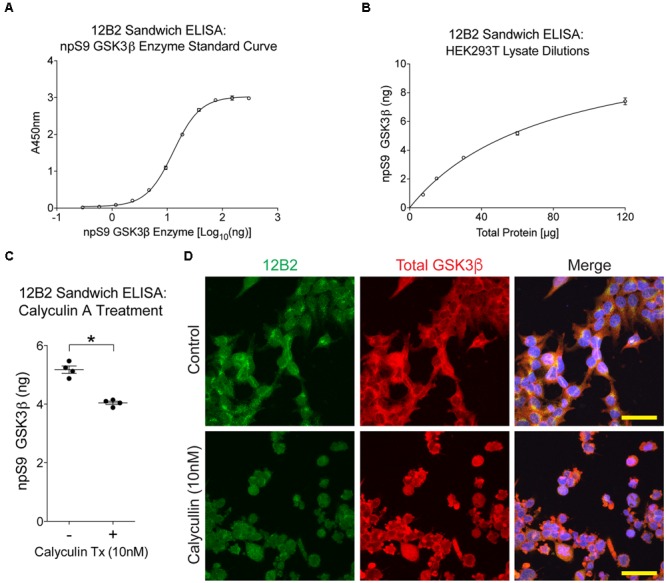
**Treating cells with protein phosphatase inhibitor decreases npS9 GSK3β in cells. (A)** A standard curve of dephosphorylated GSK3β protein captured with 12B2 antibody was used for quantitative sandwich ELISAs (*r*^2^= 0.999). **(B)** Untreated HEK293T lysates assayed in 12B2 sandwich ELISAs at 120, 60, 30, 15, and 7.5 μg total protein/well produces a linear dose response curve (*r*^2^= 0.988). Interpolation using the standard curve in **(A)** indicates that the lysate samples contain 7.4, 5.2, 3.5, 2.0, and 0.9 ng of npS9 GSK3β, respectively. **(C)** HEK293T cells were either untreated (-) or treated with 10 nM calyculin A for 30 min (+) to reduce npS9 GSK3β levels (*n* = 4 independent experiments). The lysates were used in 12B2 sandwich ELISAs. A significant reduction in npS9 GSK3β levels was detected in calyculin A (10 nM) treated cells compared to untreated cells (^∗^*p* < 0.05, unpaired *t*-test, two-tailed). The amount of npS9 GSK3β was quantitatively determined by interpolation using the recombinant GSK3β standard curve in **(A)**. **(D)** Immunofluorescence for 12B2 (green) showed an apparent qualitative reduction in npS9 GSK3β levels in HEK cells treated with calyculin A when compared to control cells. Cells were also stained with total GSK3α/β (red) and DAPI. Scale bar = 25 μm.

First, we used 12B2 sandwich ELISAs to quantitatively measure the amount of npS9 GSK3β in these lysate samples. A recombinant npS9 GSK3β standard curve (*r*^2^= 0.999) was used to interpolate the amount of npS9 GSK3β bound from the lysates (**Figure 8A**). The 12B2 sandwich ELISAs were confirmed to linearly detect npS9 GSK3β in HEK cell lysates by applying 120, 60, 30, 15, and 7.5 μg of total lysate protein, which produced a linear response curve (*r*^2^= 0.988) and 7.4, 5.2, 3.5, 2.0, and 0.9 ng of npS9 GSK3β was detected (**Figure 8B**). The 12B2 sandwich ELISAs were then used to quantitatively measure the amount of npS9 GSK3β following calyculin A treatment. A significant reduction in the amount of npS9 GSK3β [*t*(3) = 6.43, *p* = 0.008] occurred after calyculin A treatment. Based on the recombinant npS9 GSK3β standard curve the control samples contained 5.2 ± 0.12 ng and treated samples contained 4.0 ± 0.11 ng npS9 GSK3β (per 60 μg total protein; **Figure 8C**). The reduction in npS9 GSK3β levels was further confirmed by the qualitative reduction of 12B2 reactivity in HEK293T cells treated with calyculin A (**Figure 8D**).

**FIGURE 9:**
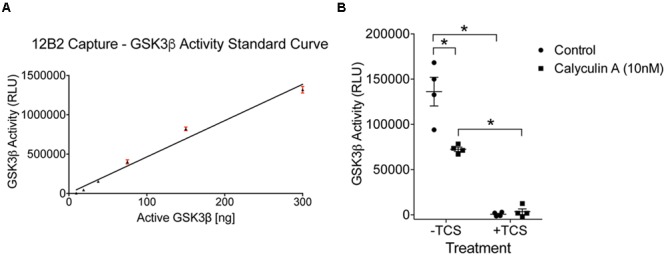
**Protein phosphatase inhibition significantly reduces GSK3β kinase activity in cells. (A)** A standard curve of active GSK3β enzyme (300 – 9.4 ng) confirmed the signal in the experimental samples was within the linear range of detection in this assay (*r*^2^= 0.97). Experiment was repeated three times. **(B)** Calyculin A treated cells showed a significant reduction in GSK3β kinase activity compared to control cells (the –TCS sample sets; all samples were used at 60 μg total protein/well). Interpolation from the recombinant GSK3β enzyme activity curve with known amounts of active GSK3β indicated that the control samples contained 29 ng of active GSK3β and calyculin A treated cells contained 15 ng. Addition of TCS-2002 (0.1 mM; +TCS), a potent GSK3β inhibitor, completely blocked kinase activity in control and calyculin A treated cells (^∗^*p* < 0.05, two-way ANOVA with Holm-Sidak *post hoc* test, two-tailed). Note that the same lysate samples used here were used in **Figure 8**. This experiment was repeated four times.

To determine whether protein phosphatase treatment caused reductions in GSK3β kinase activity, we ran the same lysates in a kinase activity assay using 12B2 to capture GSK3β from the samples. Serial dilution of recombinant GSK3β enzyme produced a linear signal (*r*^2^= 0.97; **Figure 9A**), and all experimental lysate samples were within the linear range. The activity of GSK3β was significantly reduced in calyculin A treated cells compared to control cells [calyculin A treatment factor: *F*_(1,12)_ = 13.84, *p* = 0.003; TCS treatment factor: *F*_(1,12)_ = 156.5, *p* < 0.0001; interaction factor: *F*_(1,12)_ = 16.59, *p* = 0.002; **Figure 9B**]. Interpolation from the recombinant GSK3β enzyme activity curve with known amounts of GSK3β (**Figure 9A**) indicates that the control samples contained 29 ng active GSK3β and the calyculin A treated samples contained 15 ng active GSK3β (lysate samples were used at 60 μg total protein/well). Treatment with TCS-2002, the potent GSK3β inhibitor, completely blocked kinase activity confirming GSK3β produced the signal (**Figure 9B**).

### Protein Phosphatases Dephosphorylate S9/21 in GSK3β/α Independent of the Akt Pathway

**FIGURE 10:**
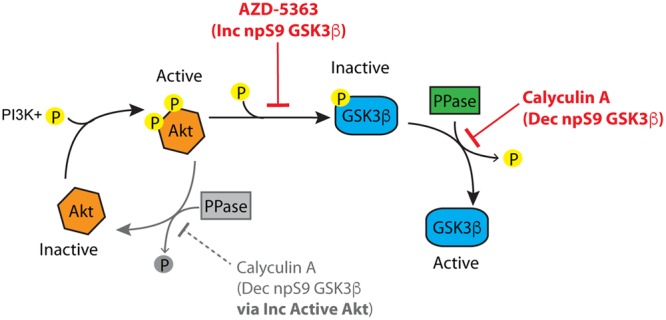
**The Akt-protein phosphatase signaling pathway involved in regulating GSK3β phosphorylation.** Active Akt (i.e., phosphorylated) inactivates GSK3β by phosphorylation at S9. Protein phosphatases can modulate GSK3β phosphorylation at S9 via two routes. (1) Protein phosphatases inactivate Akt by dephosphorylation, and (2) protein phosphatases activate GSK3β by directly dephosphorylating S9. Inhibition of Akt (with inhibitors such as AZD-5363) increases non-phosphorylated GSK3β by suppressing Akt-mediated phosphorylation of GSK3β. Inhibition of protein phosphatases (with inhibitors such as calyculin A) causes a decrease in non-phosphorylated GSK3β through the Akt pathway by increasing active Akt (the grayed portion of the Akt cycle). Protein phosphatase inhibition also leads to decreased non-phosphorylated GSK3β independent of Akt by directly dephosphorylating S9 in GSK3β. If an Akt inhibitor is applied followed by a protein phosphatase inhibitor the Akt-independent pathway can be evaluated.

**FIGURE 11:**
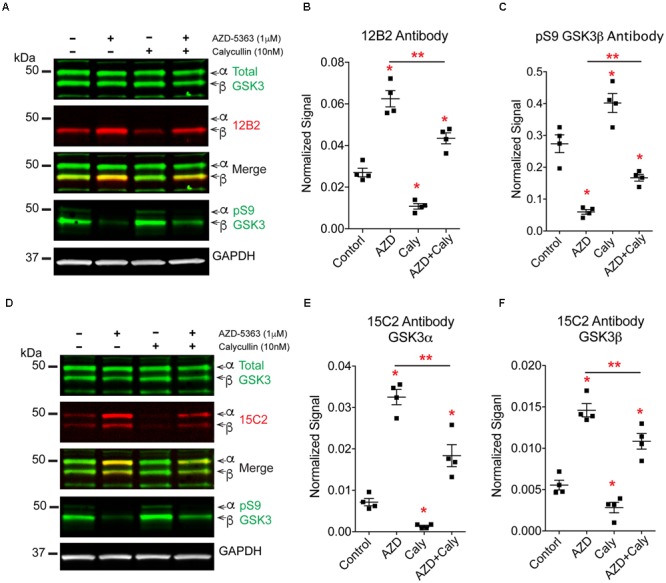
**Protein phosphatases regulate GSK3β phosphorylation independent of Akt signaling.** HEK293T cells were treated with an Akt inhibitor (AZD-5363, 1 μM), a protein phosphatase inhibitor (calyculin A, 10 nM) or the Akt inhibitor followed by the phosphatase inhibitor. Four independent experiments were run. **(A)** Western blots of samples were probed with 12B2 (npS9-GSK3β specific), total GSK3α/β, pS9-GSK3β and GAPDH (loading control). **(B)** Quantitation of the blots shows that inhibition of Akt (AZD) significantly increased npS9 GSK3β, while inhibition of protein phosphatases (Caly) significantly reduced npS9 GSK3β. When Akt signaling was blocked first and then the phosphatase inhibitor was applied (AZD + Caly) a significant reduction in the level of npS9 GSK3β occurred when compared to Akt inhibitor alone. **(C)** Quantitation of the pS9 GSK3β blots shows an opposite pattern where inhibition of Akt significantly decreased pS9 GSK3β, while inhibition of phosphatases significantly increased pS9 GSK3β. When Akt signaling was blocked and then the phosphatase inhibitor was applied a significant increase in the level of pS9 GSK3β occurred when compared to Akt treatment alone. **(D)** Western blots of samples were probed with 15C2 (npS9-GSK3α/β specific), total GSK3α/β, pS9-GSK3β and GAPDH (loading control). **(E,F)** Quantitation of the blots shows that inhibition of Akt significantly increased npS9 GSK3α and β, while inhibition of protein phosphatases significantly reduced npS9 GSK3α and β. When Akt signaling was blocked and then the phosphatase inhibitor was applied a significant reduction in the level of npS9 GSK3β and npS21 GSK3α occurred when compared to Akt inhibitor alone. Collectively, these results suggest protein phosphatases dephosphorylate Ser9/21 independent of Akt signaling. All bands are normalized to GAPDH. All groups were statistically significant from the others in (**B,C,E,F)**, but only ^∗^*p* < 0.05 vs. control and ^∗∗^*p* < 0.05 vs. AZD alone are indicated to simplify the graph and highlight the critical comparisons (one-way ANOVA, Holm-Sidak *post hoc* test). Total GSK3α/β levels, total Akt and phospho-Akt immunoblots from these experiment are displayed in **Supplementary Figure S5**.

To further define the mechanisms of protein phosphatase-mediated regulation of GSK3 we explored whether protein phosphatases modify S9/21 independent of the Akt pathway (**Figure 10**). Treatment of HEK293T cells with AZD-5363 (1 μM), an Akt inhibitor (Davies et al., 2012; Li et al., 2013), caused a robust increase in npS9 GSK3β as detected with 12B2 [**Figures 11A,B**; *F*_(3,12)_ = 69.97, *p* < 0.0001] and an increase in both npS GSK3α and β as detected with 15C2 [**Figures 11D–F**; *F*_(3,12)_ = 67.17, *p* < 0.0001] when compared to controls. As expected, this indicates that blocking Akt activity leads to the accumulation of npS GSK3. Treatment of cells with calyculin A (10 nM) caused a significant reduction in npS9 GSK3β as detected with 12B2 (**Figures 11A,B**) and a reduction in both npS GSK3α and β as detected with 15C2 (**Figures 11D–F**) when compared to controls. This confirms that inhibiting phosphatase activity allows phospho-S9/21 GSK3 to accumulate, but this can occur through two pathways because phosphatases can directly dephosphorylate both Akt (increasing phospho-Akt levels) and GSK3 (decreasing nonphospho-GSK3 levels) (**Figure 10**). To establish whether protein phosphatases dephosphorylate GSK3 at S9/21 independent of the Akt pathway, we first blocked Akt activity with AZD-5363 (for 1 h) and then calyculin A was added (for 30 min) to inhibit protein phosphatases. This treatment paradigm produced a significant reduction in npS9 GSK3β as indicated by 12B2 and both npS GSK3α and β as detected by 15C2 (**Figures 11D–F**) when compared to AZD-5363 alone. Furthermore, we observed the opposite result when blots were probed with a pS9 GSK3β antibody [**Figure 11C**, *F*_(3,12)_ = 46.79, *p* < 0.0001]. The AZD-5363 alone treatment resulted in increased active Akt levels [i.e., pT308 and pS473 Akt; **Supplementary Figures S5A–C**, pT308: *F*_(3,12)_ = 20.13, *p* < 0.0001; pS473: *F*_(3,12)_ = 7.699, *p* = 0.004] and increased npS GSK3 levels demonstrating that we effectively blocked Akt activity at the dose used (ineffective inhibition would lead to reduced npS GSK3 and increased pS GSK3 in the presence of elevated active Akt levels). It is noteworthy that neither total GSK3α/β [Total α: *F*_(3,12)_ = 1.824, *p* = 0.20; Total β: *F*_(3,12)_ = 0.926, *p* = 0.46] nor total Akt levels [*F*_(3,12)_ = 0.955, *p* = 0.45] were significantly affected with these treatments (**Supplementary Figures S5D,E**).

## Discussion

The GSK3β enzyme is one of the most widely studied S/T kinases because of its role in several biological processes (Kim and Kimmel, 2006; Kockeritz et al., 2006; Forde and Dale, 2007; Hur and Zhou, 2010; Medina and Wandosell, 2011; Beurel et al., 2015) and disease states (Hernandez and Avila, 2008; Cadigan and Peifer, 2009; Golpich et al., 2015; Lal et al., 2015; Li et al., 2015; O’Leary and Nolan, 2015). Not surprisingly, GSK3β regulation is tightly controlled by several mechanisms including phosphorylation, substrate priming, truncation, protein complex association and subcellular localization (Medina and Wandosell, 2011). Phosphorylation is the most prominent and well-understood regulatory mechanisms, and phosphorylation of S9 in GSK3β (S21 in GSK3α) is a dominant negative regulatory mechanism because the pS9 region competitively blocks substrate docking by mimicking primed substrates. In general, when S9 is not phosphorylated, the enzyme is typically considered “active” because other modifications such as phosphorylation of tyrosine 216 (or tyrosine 276 in GSK3α) appear to occur at near stoichiometric levels and during translation in a chaperone-dependent mechanism (Hughes et al., 1993; Wang et al., 1994a; Cohen and Goedert, 2004; Cole et al., 2004). However, there are other Ser/Thr residues in GSK3β, such as T43, T390 and S389, that are targets of other kinases (i.e., Erk and/or p38 MAPK) and modulate the activity of GSK3β as well (Ding et al., 2005; Thornton et al., 2008). Thus, levels of npS9 GSK3β can generally be a useful surrogate marker for the amount of GSK3 in an “active-state,” and here we show that 12B2 or 15C2 reactivity in western blots correlates well with kinase activity (at least using recombinant proteins *in vitro*). However, the npS GSK3 antibodies do not directly speak to kinase activity levels and GSK3β activity should be directly assayed when possible. To this end, we demonstrate that 12B2 can be applied in assays that allow direct measurement of GSK3β kinase activity in experimental lysates, and that the same reagent directly measures npS9 GSK3β in several assay formats.

### Considerations for Producing Stable, High-Quality Monoclonal Antibodies

Our understanding is that npS9 GSK3β reagents were difficult to obtain by other groups and companies. We used a relatively unique immunization approach by combining a normal peptide, a tandem repeat peptide and an arginine enantiomer peptide. The tandem peptide provides more antigens specifically centered on the residue of interest, while arginine enantiomers are known to enhance antigenicity (Benkirane et al., 1993; Guichard et al., 1994). We rule out antibodies that react against KLH, neoepitopes in the tandem peptide and the D-arginine by using normal GSK3β peptides not conjugated to KLH in all screening assays. Moreover, we place a great deal of importance on identifying specificity early in the process. For example, assessing differences in reactivity for npS9 GSK3β, pS9 GSK3β and npS21 GSK3α begins at the fusion stage. We also require relatively robust serum titer signal (1:25,000 or greater dilution), high levels of reactivity in subsequent ELISAs (absorbance >1.0), stability after repeated freeze/thaw cycles, and mycoplasma negativity to consider a line stable and of sufficient quality. Typically, we subclone lines 2–3 times every time we retrieve a clone from long-term storage to ensure clone stability and consistency. Overall, these criteria help establish high quality, sustainable lines for producing reagents indefinitely.

### Advantages of the New GSK3 Antibody Reagents

Despite the importance of GSK3 and widespread interest in GSK3, reagents for directly measuring npS9/21 GSK3β/α (i.e., the so-called “active” form) did not exist. Currently available antibodies allow only indirect assessment of changes in GSK3 activation by looking at the phosphorylated N-terminal serine residues and many of them lack a high degree of specificity. We provide rigorous, detailed characterization of two novel monoclonal antibodies. The 12B2 antibody specifically detects npS9 GSK3β, and lacks reactivity when S9 is phosphorylated and does not react with GSK3α proteins. The 15C2 antibody works similarly with GSK3β but also detects npS21 GSK3α making it useful for also studying GSK3α regulation. It is noteworthy that neither of these antibodies showed detectable reactivity against phospho-S9/21 peptides in ELISAs (even when high antibody concentrations or large amounts of peptides were used) or against *in vitro* phosphorylated recombinant GSK3 in western blotting (up to 300 ng protein). Evaluating total GSK3β levels is not required with these new reagents when equivalent samples are used, but this may remain a valuable assessment if determining whether experimental conditions alter both the amount of npS9 GSK3β and total GSK3β is desired. All of the reagents detect GSK3 enzymes in human, mouse and rat, as well a number of commonly used human, mouse and rat cell types, which is expected considering the high homology across these species. The high sequence homology in this region goes across many species (both vertebrates and invertebrates) (Forde and Dale, 2007), which likely expands the usefulness of these reagents.

The fact that these antibodies work in several assays further highlights their advantages. We tested these antibodies in indirect ELISAs, western blotting, immunoprecipitations, cell culture ICF, and tissue section IHC using a range of samples including synthetic peptides, recombinant GSK3β and α, as well as human and rodent cells and tissues. Providing npS9 GSK3-specific reagents will allow researchers versatility and the added benefit of using the same reagents in several assay formats. The fact that these antibodies work in both biochemical assays and immunostaining assays in cultured cells and tissue sections represents another advantage because identifying subcellular localization of changes in npS9 GSK3β can be directly related to changes in protein levels and kinase activity. Interestingly, the immunofluorescence studies in cultured cells showed a predominance of punctate staining with npS9 GSK3β antibodies (particularly 12B2), which may represent signalosomes or other multi-component complexes containing npS9 GSK3β enzymes (Bilic et al., 2007; Cadigan and Peifer, 2009). In fact, the siRNA studies clearly show that these puncta are reduced in cells, confirming they contain npS9 GSK3β. Thus, the reagents described here provide new all-in-one reagents for directly measuring npS9 GSK3β and GSK3β kinase activity levels that exhibit great assay versatility, subcellular localization and cross-species utilization (**Table 1**).

**Table 1:** Summary of GSK3β antibody performance in assays tested in the current work.

Antibody (Isotype)	Peptide Indirect ELISA	Rec Prot WB	Brain Lysate WB	Lysate IP	Rec Prot sELISA	Lysate sELISA	IHC (rat brain)	IHC (human brain)	ICF (culture)	WB (activity assay samples)	Kinase Activity Assay
12B2 (IgG1)	β > > α	β only	β only	Yes (β)	++	Yes *r*^2^= 0.99	+++	+++	++	*r*^2^= 0.92 *p* < 0.0001	Yes
15C2 (IgG1)	β < α	β < α	β < α	Yes (α/β)	–	nd	+++	+++	++	*r*^2^= 0.90 *p* < 0.0001	nd

*– poor or no signal, + weak signal, ++ moderate signal, +++ strong signal and ++++ best signal relative to other antibodies. Rec Prot, recombinant protein; ELISA, enzyme linked immunosorbent assay; WB, western blot; IP, immunoprecipitation; sELISA, sandwich ELISA; IP, immunoprecipitation; IHC, immunohistochemistry; ICF, immunocytofluorescence; nd, not determined.*

The most compelling data supporting the use of these antibodies to provide biological insights are those confirming that they directly measure, in a linear fashion, the amount of npS9 GSK3β. We demonstrate that these reagents detect changes in the amount of npS9 GSK3β and that the signals on immunoblots correlate very well with the kinase activity of GSK3β using recombinant proteins and experimentally induced GSK3β inhibition (e.g., calyculin treated cells). Moreover, the use of a recombinant protein standard curve in the sandwich ELISAs and the kinase activity assays allows quantitation of unknown amounts of active GSK3β and GSK3β activity levels in whole cell lysates. A few approaches are currently used to measure GSK3β activity in experimental lysate samples that often involve combining immunoprecipitation of total GSK3β and *in vitro* GSK3β activity assays (Welsh et al., 1997; Bijur and Jope, 2001; Bowley et al., 2005). Thus, these reagents have a distinct advantage over currently available reagents because other reagents do no target the npS9 (or “active”) form of GSK3β, and subsequently the new antibodies enhance our ability to study GSK3 regulation.

The performance of each antibody was remarkably similar across assays, but some differences were observed. For instance, the synthetic peptide indirect ELISAs showed moderate affinity differences between GSK3β and GSK3α with 12B2, while 15C2 showed a stronger reaction with GSK3α over GSK3β peptides. However, assays that used the full-length protein showed the differential affinity for GSK3β and α was substantial for 12B2, and 15C2 reacted equally as well with both forms of GSK3. Once lysate samples were used, the differential reactivity between GSK3β and GSK3α were quite robust with no 12B2 reactivity for GSK3α under the conditions used. Overall, 12B2 produced results demonstrating it is specific to npS9 GSK3β isoform, while 15C2 is specific to npS9 GSK3β and npS21 GSK3α.

### Novel GSK3β Antibodies Demonstrate that Protein Phosphatases Reduce npS9 GSK3β Levels and Kinase Activity through an Akt-Independent Pathway

Protein phosphatases are thought to regulate GSK3β activity through dephosphorylation of S9, where increased phosphatase activity leads to increased GSK3β activity, and likewise, protein phosphatase inhibition causes GSK3β inhibition by reducing nonphospho-S9 GSK3β. Indeed, this regulatory pathway appears to play a role in GSK3β activation under several biological contexts, including cell excitability, survival of leukemic progenitors, insulin signaling, growth factor signaling, and motor protein regulation, among others (Sutherland et al., 1993; Leung-Hagesteijn et al., 2001; Morfini et al., 2004; Lee et al., 2005; Szatmari et al., 2005; Bertrand et al., 2012; Ma et al., 2012). However, protein phosphatases also may influence GSK3β phosphorylation through the Akt pathway (Millward et al., 1999; Bononi et al., 2011). In the Akt pathway, stimulation of the phosphoinositide 3-kinase/Akt pathway leads to activation of Akt via phosphorylation of T308 and S473 residues, and then active Akt phosphorylates GSK3β/α at S9/21 (Gold et al., 2000; Varea et al., 2010; Majewska and Szeliga, 2016). Protein phosphatases (in particular protein phosphatase 2A) dephosphorylate Akt leading to Akt inactivation, and subsequently increased active GSK3β/α via the loss of Akt-mediated S9/21 phosphorylation (Millward et al., 1999; Bononi et al., 2011).

Previous studies were unable to directly demonstrate that protein phosphatases-mediated effects were due to changes in the npS9 GSK3β because such reagents did not exist. Furthermore, we set out to determine whether protein phosphatases dephosphorylate GSK3β/α at S9/S21 independent of Akt signaling. Our results using both 12B2 and 15C2 suggest that protein phosphatases decrease npS9/21 GSK3β/α levels via direct dephosphorylation that is independent of the Akt pathway. As expected, blocking Akt activity robustly increased levels of npS GSK3 because Akt could no longer phosphorylate GSK3, despite an elevation in active Akt levels (i.e., pT308 and pS473 Akt). When Akt was inhibited to eliminate contributions of the Akt pathway prior to application of protein phosphatase inhibitor, there was a significant reduction in npS GSK3 and increase in pS GSK3 suggesting that GSK3 dephosphorylation by protein phosphatases occurs independent of Akt pathway. We also show that the reduced levels of npS9 GSK3β in HEK293 cells treated with protein phosphatase inhibitor causes a reduction in GSK3β kinase activity by using the 12B2 npS9 GSK3β antibody. In fact, the same reagent was used to validate this biologically relevant mechanism of GSK3β regulation across several assay formats, including western blots, ELISAs, immunocytofluorescence and kinase activity assays. Thus, these reagents show strong inter-assay validation and should provide researchers the ability to directly and more accurately study GSK3β regulation and activity across many biological contexts.

## Conclusion

The lack of reagents that specifically and directly assess the level of npS9/21 GSK3β/α has burdened the research community. Here, we provide the characterization of one monoclonal antibody that specifically reacts with GSK3β when S9 is not phosphorylated, as well as one antibody that also reacts with GSK3α when S21 is not phosphorylated. Notably, these reagents will allow researchers to directly measure the level of npS GSK3 in experimental conditions using a wide range of assay formats. We provide evidence that these reagents will help researchers study GSK3β regulation in biological systems by demonstrating that protein phosphatase inhibition leads to reduced GSK3β activity via reductions in amount of npS9 forms of GSK3β independent of Akt signaling. Collectively, these reagents should provide significant advantages over existing reagents and will help to facilitate GSK3β research across many fields of biology. Finally, if changes in GSK3β activity are an important marker for a particular disease state, which is likely considering the involvement of GSK3β in several disease processes, these reagents may be extremely useful in developing diagnostic and/or biomarker assays for diseases.

## Author Contributions

TG and NK worked together on all aspects of this work and both approve the final version of the manuscript.

## Conflict of Interest Statement

The authors declare that the research was conducted in the absence of any commercial or financial relationships that could be construed as a potential conflict of interest.

## Supplementary Material

FIGURE S1 **Hybridoma fusion and subcloning procedures. (A–C)** Indirect ELISA titer of serum from immunized animals after the 3rd **(A)** and 6th **(B)** immunization boost. Animal T10 and N00 were used for fusions because they produced the strongest reactivity after the 3rd and 6th boosts, respectively. **(C,D)** The fusion cultures were screened against npS9 GSK3β, pS9 GSK3β and npS21 GSK3α peptides. ^∗^indicates reactivity with npS9 much greater than npS21 GSK3α and no reactivity with pS9 GSK3β; ^∗∗^indicates reactivity with more npS21 GSK3α than npS9 GSK3β and no reactivity with pS9 GSK3β. The 12B2 **(C)** and15C2 **(D)** cultures were continued to the first subclone. Subsequent subclone cultures were similarly screened against these peptides in indirect ELISAs (using same method) to evaluate specificity during the cloning process (data not shown). We typically require that the percent of reactive clone wells should be ≥95% by the third subclone (12B2 = 99% and 15C2 = 100%). **(E)** Phosphorylation at serine 9 of the pS9 GSK3β peptide was confirmed using a pS9 GSK3β-specific antibody in indirect ELISAs.

FIGURE S2 **12B2 and 15C2 label npS GSK3 isoforms in multiple cell types. (A)** Cell lysates from SH-SY5Y neuroblastoma cells (human), HEK293T cells (human), primary neurons (rat), U373 glioblastoma cells (human), and Neuro-2a neuroblastoma cells (mouse, N2a) were probed with total GSK3α/β (green) and 12B2 (red) antibodies to detect npS9 GSK3β. Much like the brain lysates in **Figure 3**, 12B2 specifically labels only npS9 GSK3β in all cell types, but varying amounts were detected in the difference cells (all loaded at 50 μg/lane). **(B)** Cell lysates from the same cells were probed with total GSK3α/β (green) and 15C2 (red) antibodies to detect npS9/21 GSK3β/α. Much like the brain lysates in **Figure 3**, 15C2 specifically labels both npS9 GSK3β and npS21 GSK3α in all cell types, but varying amounts were detected in the difference cells (all loaded at 50 μg/lane).

FIGURE S3 **Primary delete controls for cell culture immunofluorescence and tissue immunohistochemistry. (A)** HEK293T cells were processed for immunofluorescence with the exception that the npS9 GSK3β (green channel) and total GSK3 α/β (red channel) primary antibodies were omitted (cells were counterstained with DAPI, blue channel). A lack of staining confirms that the signals were due to reactivity with the primary antibodies and not artifacts from other components of the staining procedure or imaging cells with fluorescence. **(B)** Rat and human tissue sections were processed for immunohistochemistry with the exception that the npS9 GSK3β antibodies were omitted. A lack of staining confirms that the signals were due to reactivity with the primary antibody and not artifacts from processing tissue through the staining procedure. All scale bars in **(A,B)** are 50 μm.

FIGURE S4 **Effects of GAPDH siRNA in HEK293T Cells.** HEK293T cells were treated with GAPDH siRNAs and probed with 12B2 or 15C2 and total GSK3β/α antibodies (see **Figures 5A** and **6A** for blot images). **(A)** Quantification shows that GSK3β siRNA caused a reduction of 82% in signal for the GAPDH when compared to control cells. **(B)** Quantification of the GSK3α and GSK3β bands with 15C2 (which labels both npS9 GSK3β and npS21 GSK3α) showed only minimal changes compared to controls (-17%).

FIGURE S5 **Akt inhibitor and protein phosphatase inhibitor treatments affect Akt phosphorylation, but not total Akt or GSK3 levels.** HEK293T cells were treated with an Akt inhibitor (AZD-5363, 1 μM), a protein phosphatase inhibitor (calyculin A, 10 nM) or the Akt inhibitor followed by the phosphatase inhibitor to demonstrate the potential utility of 12B2 and 15C2 in studying GSK3 regulation. Four independent experiments were run (same as **Figure 11**). **(A)** Western blots of samples were probed with pT308 Akt and pS473 Akt (active phospho-Akt), total Akt and GAPDH (loading control). **(B,C)** Quantitation of the blots shows that inhibition of Akt (AZD) significantly increased both **(B)** pT308 and **(C)** pS473 Akt. The fact that AZD caused upregulation of npS9/21 GSK3β/α (see **Figure 11**) and increased active phospho-Akt (which would normally decrease npS GSK3 levels) confirms the effectiveness of the AZD dose. **(D,E)** None of the treatments significantly affected the levels of **(D)** total Akt (*p* = 0.45), as well as **(E)** total GSKα (*p* = 0.20) or total GSK3β (*p* = 0.46). All bands are normalized to GAPDH. (^∗^*p* < 0.05 vs. control; one-way ANOVA, Holm-Sidak *post hoc* test).
