# Supplementary figures and images for: Novel Non-phosphorylated Serine 9/21 GSK3β/α Antibodies: Expanding the Tools for Studying GSK3 Regulation
Source: Front Mol Neurosci. 2016 Nov 17;9:123. doi: 10.3389/fnmol.2016.00123 (PMC5112268; doi:10.3389/fnmol.2016.00123)

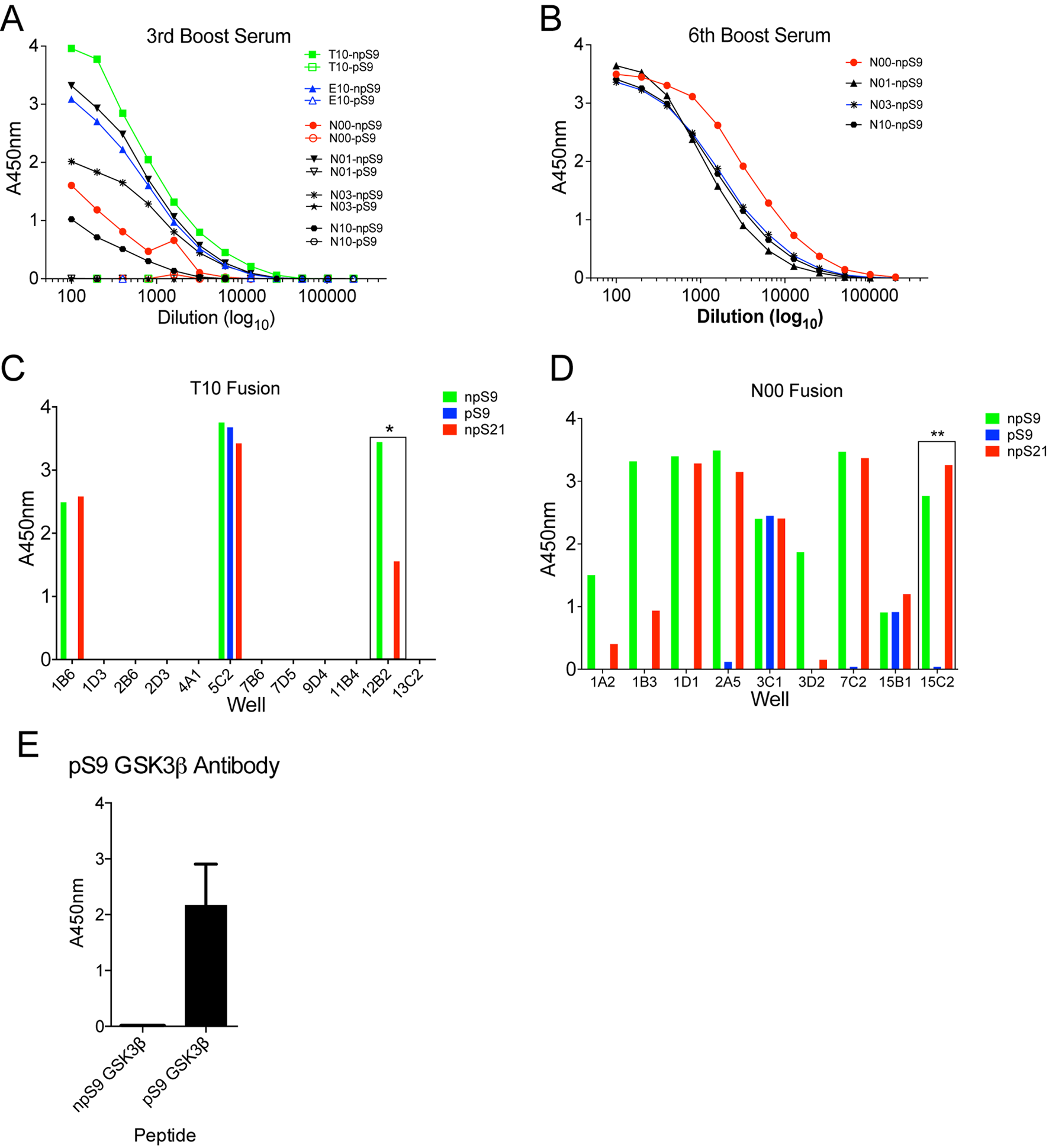

Supplement: FIGURE S1 — Hybridoma fusion and subcloning procedures. (A–C) Indirect ELISA titer of serum from immunized animals after the 3rd (A) and 6th (B) immunization boost. Animal T10 and N00 were used for fusions because they produced the strongest reactivity after the 3rd and 6th boosts, respectively. (C,D) The fusion cultures were screened against npS9 GSK3β, pS9 GSK3β and npS21 GSK3α peptides. ∗indicates reactivity with npS9 much greater than npS21 GSK3α and no reactivity with pS9 GSK3β; ∗∗indicates reactivity with more npS21 GSK3α than npS9 GSK3β and no reactivity with pS9 GSK3β. The 12B2 (C) and15C2 (D) cultures were continued to the first subclone. Subsequent subclone cultures were similarly screened against these peptides in indirect ELISAs (using same method) to evaluate specificity during the cloning process (data not shown). We typically require that the percent of reactive clone wells should be ≥95% by the third subclone (12B2 = 99% and 15C2 = 100%). (E) Phosphorylation at serine 9 of the pS9 GSK3β peptide was confirmed using a pS9 GSK3β-specific antibody in indirect ELISAs. [file Image_1.TIF]

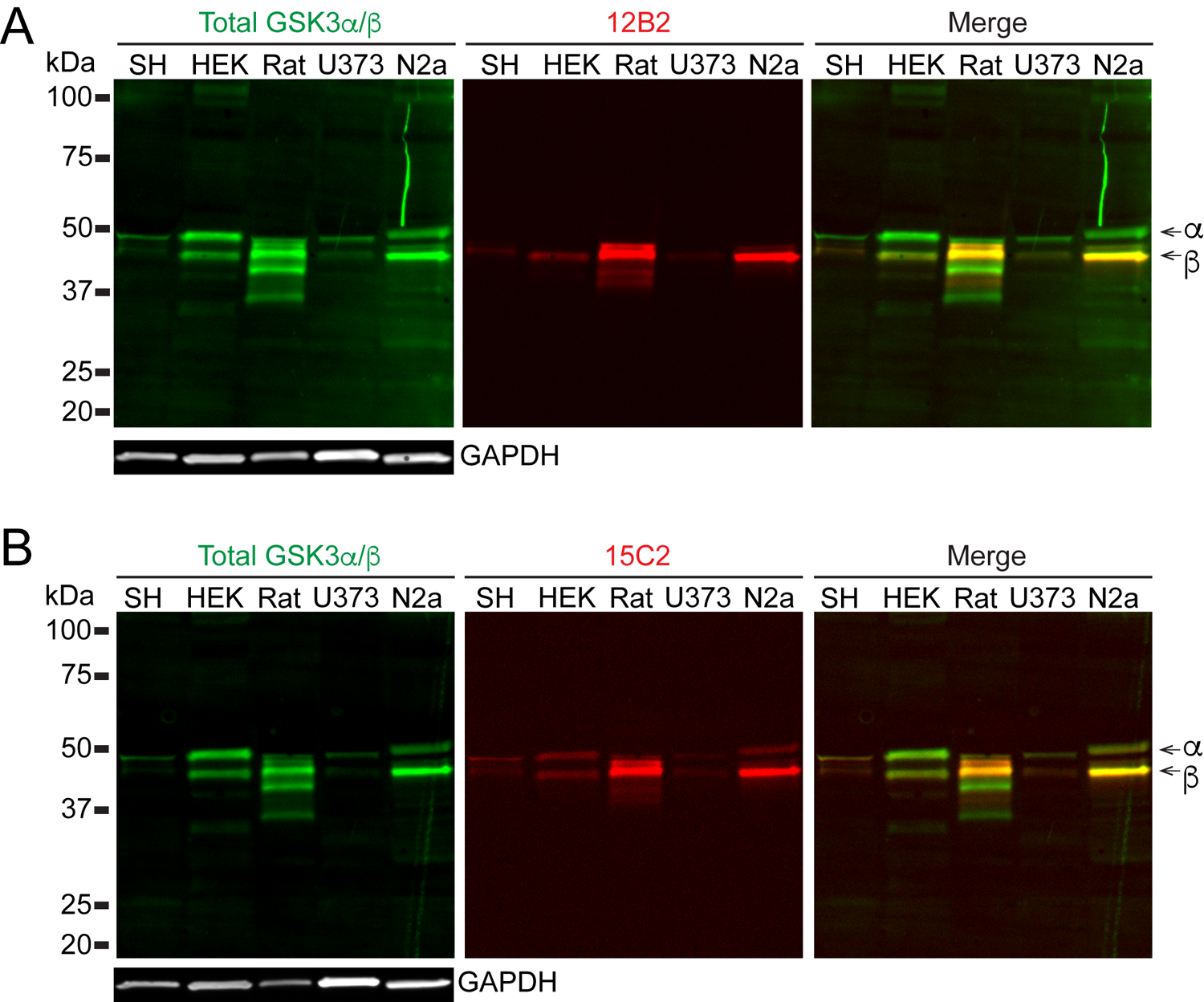

Supplement: FIGURE S2 — 12B2 and 15C2 label npS GSK3 isoforms in multiple cell types. (A) Cell lysates from SH-SY5Y neuroblastoma cells (human), HEK293T cells (human), primary neurons (rat), U373 glioblastoma cells (human), and Neuro-2a neuroblastoma cells (mouse, N2a) were probed with total GSK3α/β (green) and 12B2 (red) antibodies to detect npS9 GSK3β. Much like the brain lysates in Figure 3, 12B2 specifically labels only npS9 GSK3β in all cell types, but varying amounts were detected in the difference cells (all loaded at 50 μg/lane). (B) Cell lysates from the same cells were probed with total GSK3α/β (green) and 15C2 (red) antibodies to detect npS9/21 GSK3β/α. Much like the brain lysates in Figure 3, 15C2 specifically labels both npS9 GSK3β and npS21 GSK3α in all cell types, but varying amounts were detected in the difference cells (all loaded at 50 μg/lane). [file Image_2.TIF]

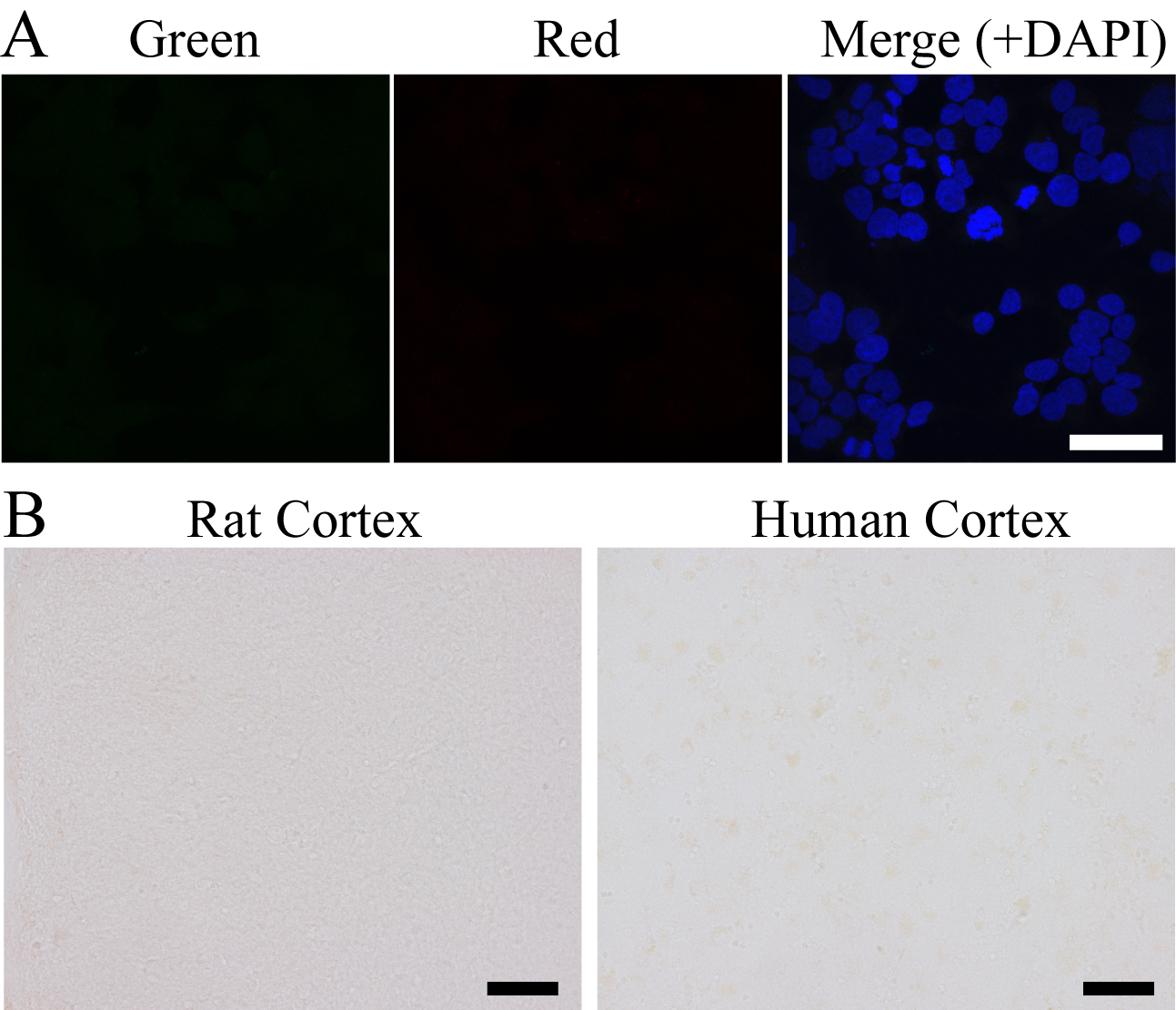

Supplement: FIGURE S3 — Primary delete controls for cell culture immunofluorescence and tissue immunohistochemistry. (A) HEK293T cells were processed for immunofluorescence with the exception that the npS9 GSK3β (green channel) and total GSK3 α/β (red channel) primary antibodies were omitted (cells were counterstained with DAPI, blue channel). A lack of staining confirms that the signals were due to reactivity with the primary antibodies and not artifacts from other components of the staining procedure or imaging cells with fluorescence. (B) Rat and human tissue sections were processed for immunohistochemistry with the exception that the npS9 GSK3β antibodies were omitted. A lack of staining confirms that the signals were due to reactivity with the primary antibody and not artifacts from processing tissue through the staining procedure. All scale bars in (A,B) are 50 μm. [file Image_3.TIF]

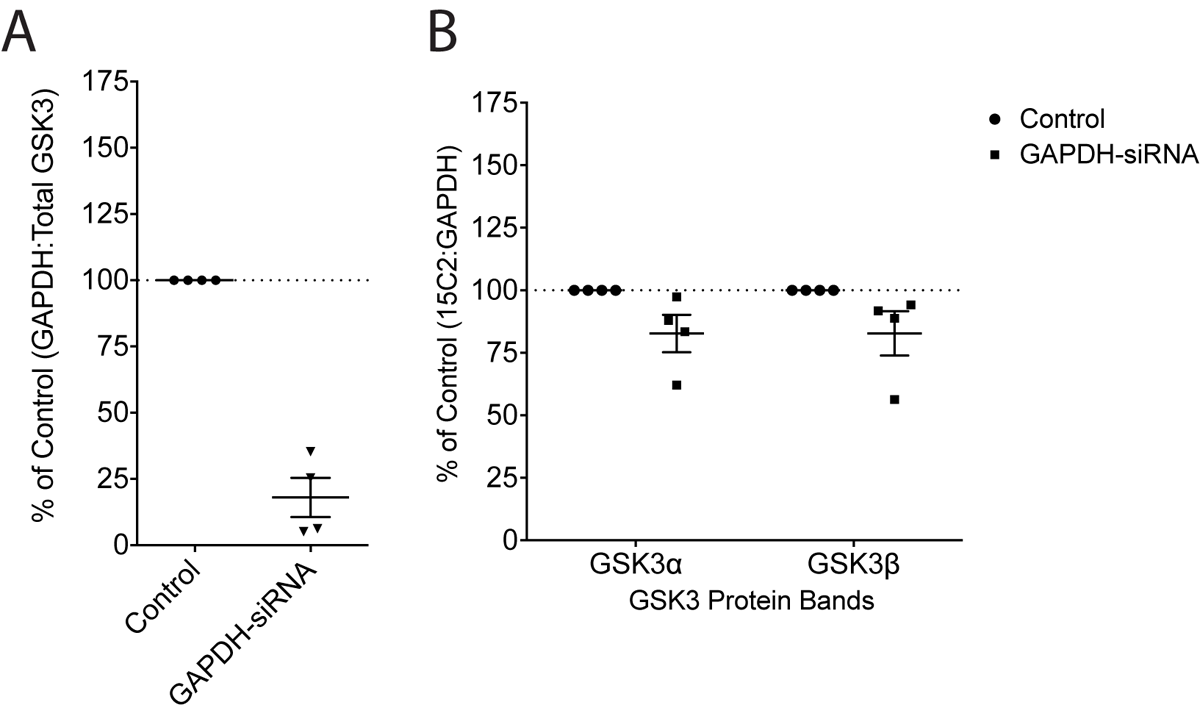

Supplement: FIGURE S4 — Effects of GAPDH siRNA in HEK293T Cells. HEK293T cells were treated with GAPDH siRNAs and probed with 12B2 or 15C2 and total GSK3β/α antibodies (see Figures 5A and 6A for blot images). (A) Quantification shows that GSK3β siRNA caused a reduction of 82% in signal for the GAPDH when compared to control cells. (B) Quantification of the GSK3α and GSK3β bands with 15C2 (which labels both npS9 GSK3β and npS21 GSK3α) showed only minimal changes compared to controls (-17%). [file Image_4.TIF]

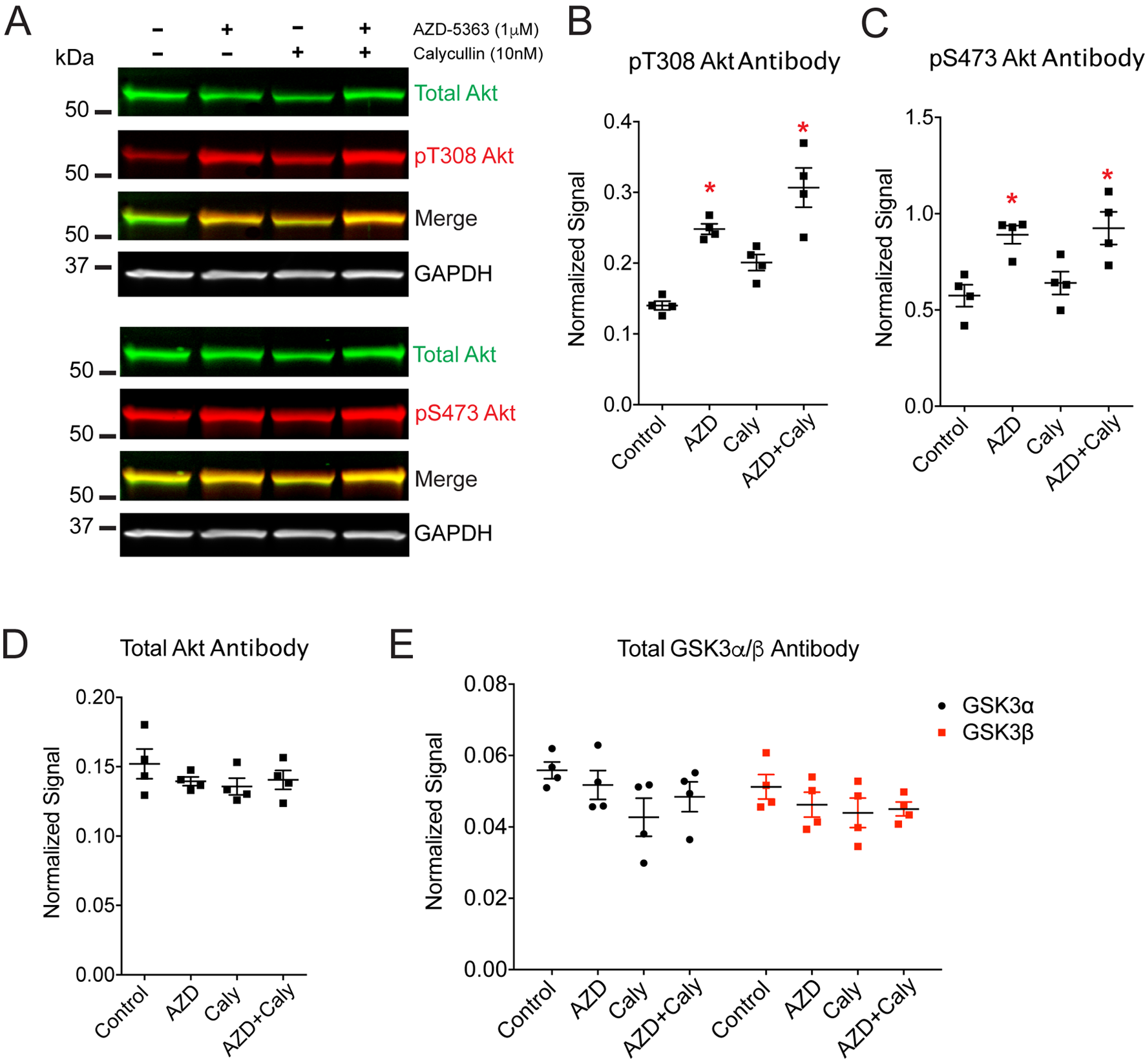

Supplement: FIGURE S5 — Akt inhibitor and protein phosphatase inhibitor treatments affect Akt phosphorylation, but not total Akt or GSK3 levels. HEK293T cells were treated with an Akt inhibitor (AZD-5363, 1 μM), a protein phosphatase inhibitor (calyculin A, 10 nM) or the Akt inhibitor followed by the phosphatase inhibitor to demonstrate the potential utility of 12B2 and 15C2 in studying GSK3 regulation. Four independent experiments were run (same as Figure 11). (A) Western blots of samples were probed with pT308 Akt and pS473 Akt (active phospho-Akt), total Akt and GAPDH (loading control). (B,C) Quantitation of the blots shows that inhibition of Akt (AZD) significantly increased both (B) pT308 and (C) pS473 Akt. The fact that AZD caused upregulation of npS9/21 GSK3β/α (see Figure 11) and increased active phospho-Akt (which would normally decrease npS GSK3 levels) confirms the effectiveness of the AZD dose. (D,E) None of the treatments significantly affected the levels of (D) total Akt (p = 0.45), as well as (E) total GSKα (p = 0.20) or total GSK3β (p = 0.46). All bands are normalized to GAPDH. (∗p < 0.05 vs. control; one-way ANOVA, Holm-Sidak post hoc test). [file Image_5.TIF]
